# Clinical, neuropathological, and immunological short‐ and long‐term feature of a mouse model mimicking human herpes virus encephalitis

**DOI:** 10.1111/bpa.13031

**Published:** 2021-10-28

**Authors:** Julia Sehl‐Ewert, Theresa Schwaiger, Alexander Schäfer, Julia E. Hölper, Barbara G. Klupp, Jens P. Teifke, Ulrike Blohm, Thomas C. Mettenleiter

**Affiliations:** ^1^ Department of Experimental Animal Facilities and Biorisk Management Friedrich‐Loeffler‐Institut Greifswald‐Insel Riems Germany; ^2^ Institute of Immunology Friedrich‐Loeffler‐Institut Greifswald‐Insel Riems Germany; ^3^ ViraTherapeutics GmbH Rum Austria; ^4^ Institute of Molecular Virology and Cell Biology Friedrich‐Loeffler‐Institut Greifswald‐Insel Riems Germany

**Keywords:** alphaherpesvirus, herpes simplex virus, herpetic encephalitis, long‐term damage, mouse model, pseudorabies virus

## Abstract

Herpes simplex encephalitis (HSE) is one of the most serious diseases of the nervous system in humans. However, its pathogenesis is still only poorly understood. Although several mouse models of predominantly herpes simplex virus 1 (HSV‐1) infections mimic different crucial aspects of HSE, central questions remain unanswered. They comprise the specific temporofrontal tropism, viral spread within the central nervous system (CNS), as well as potential molecular and immunological barriers that drive virus into latency while only rarely resulting in severe HSE. We have recently proposed an alternative mouse model by using a pseudorabies virus (PrV) mutant that more faithfully represents the striking features of human HSE: temporofrontal meningoencephalitis with few severely, but generally only moderately to subclinically affected mice as well as characteristic behavioral abnormalities. Here, we characterized this animal model using 6‐ to 8‐week‐old female CD‐1 mice in more detail. Long‐term investigation over 6 months consistently revealed a biphasic course of infection accompanied by recurring clinical signs including behavioral alterations and mainly mild meningoencephalitis restricted to the temporal and frontal lobes. By histopathological and immunological analyses, we followed the kinetics and spatial distribution of inflammatory lesions as well as the underlying cytokine expression in the CNS over 21 days within the acute phase of infection. Affecting the temporal lobes, the inflammatory infiltrate was composed of lymphocytes and macrophages showing a predominantly lymphocytic shift 15 days after infection. A strong increase was observed in cytokines CXCL10, CCL2, CCL5, and CXCL1 recruiting inflammatory cells to the CNS. Unlike the majority of infected mice, strongly affected animals demonstrated extensive temporal lobe edema, which is typically present in severe human HSE cases. In summary, these results support the validity of our animal model for in‐depth investigation of HSE pathogenesis.

## Graphical abstract

Temporofrontal meningoencephalitis in Pseudorabies virus mutant infected mice reveal striking histopathological and clinical analogies to human Herpes simplex encephalitis. Mice that are usually only moderately or subclinically affected were able to survive, although meningoencephalitis was moderate to severe in response to viral infection in the acute phase (21 days), confirmed by immunological investigation. In the later phase (beyond 21 days), the inflammatory response was mainly mild, but remained detectable up to 6 months in infected animals. A biphasic disease was clinically evident.

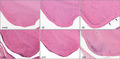

## 1 INTRODUCTION

Neurotropic alphaherpesviruses including herpes simplex viruses 1 and 2 (HSV‐1, HSV‐2) and Varicella Zoster Virus (VZV) are major human pathogens which can cause devastating neurological diseases [1]. Alphaherpesviruses typically initiate productive infections in mucosal epithelial cells. Subsequently, peripheral sensory nerve endings are infected and viral particles are transported retrogradely within their axons to ganglia of the peripheral nervous system (PNS) to establish reactivatable, life‐long latency [2]. HSV‐1 infection is the major cause of Herpes Simplex Encephalitis (HSE) [3]. HSE occurs sporadically and is characterized by high mortality of up to 70%, if undiagnosed and untreated, with only a minority of patients returning to normal life [4]. Patients initially present with unspecific clinical signs, which, however, get worse with disease progression and include disorientation, aphasia, changes in mental status, disorders of cranial nerves IV and VI, or seizures [5, 6]. Specifically, behavioral abnormalities occur which include hypomania, Kluver–Bucy syndrome, and memory impairment [7]. Survivors suffer from incriminating life‐long sequelae like speech dysfunctions, behavioral, memory and cognitive alterations, and epilepsy [8]. Apart from usually severe affection, few subacute forms of HSE have been described [9]. Further, rare cases of relapsing or chronic CNS inflammation have been reported in both immunocompetent and immunocompromised individuals [10, 11, 12].

HSE is characterized by asymmetrical necrotizing inflammation which is mainly restricted to the temporal as well as to the frontal lobe and insular cortex [13, 14]. Macroscopically, brains of surviving HSE patients reveal predominantly unilateral atrophy and yellow‐brownish discoloration resulting from necrosis and microhemorrhages of affected brain areas [15, 16]. Histologically, destruction of grey and white matter, necrosis of cortical neurons, glial activation as well as leptomeningeal, and scattered parenchymal infiltration with lymphocytes and histiocytes are present [14, 15, 17, 18]. Extensive edema has been reported [14, 18], whereas glial nodules are frequently detected [17]. Extra temporal involvement of HSE has been described in more than half of the cases and includes lesions in the frontal and parietal cortex, occipital lobe, basal ganglia, brain stem, and pons [19]. Intranuclear inclusion bodies are only inconsistently observed in neurons and astrocytes [14, 17, 20]. Rather rare reports include calcification within necrotic brain areas [21] or granulomatous inflammation with foci of mineralization [22]. Viral antigen containing neurons and astrocytes appear predominantly in amygdaloid nuclei, cortex and white matter of the lateral olfactory striae, entorhinal cortex, subiculum, hippocampus, insula, and cingulate gyrus and to a lesser extent in olfactory bulbs and pons [18].

Despite decades of intensive research, major questions regarding the pathogenesis of HSE remain unanswered. Of central importance is the elucidation of factors enabling HSV‐1 to traverse the peripheral nervous system for access to the central nervous system (CNS), specifically to the temporal lobe. Moreover, it is unclear why HSV‐1 infection leads to fatal encephalitis in some individuals, but normally results in life‐long latency without any clinical signs. In addition, subclinical HSE with recurring reactivation events and associated psychiatric disorders has been discussed decades ago [23, 24]. Valuable knowledge has been gained from a variety of animal models to better understand pathogenesis of the disease, although the data are quite heterogeneous because of the large number of different virus strains, inoculation routes, and variable genetic backgrounds of the animals [25]. In this context, especially HSV‐1‐infected mice fail to reflect crucial elements of human encephalitis appropriately, because mice develop brain stem or cerebellar encephalitis rather than inflammation associated with the temporal lobe. Although mice are generally highly susceptible to HSV‐1 infection, disease outcomes as well as mortality differ widely between inbred mouse strains [26]. Highly susceptible mice usually develop severe neurological signs and succumb to death shortly after infection [27], preventing a more detailed investigation of long‐term damage and lesions associated with behavioral abnormalities as seen in human patients.

We have recently reported on an animal model for HSE which revealed striking analogies to human disease [28]. In our study, 6‐ to 8‐week‐old female CD‐1 mice developed marked meningoencephalitis after intranasal infection with a mutant of the neurotropic alphaherpesvirus pseudorabies virus (PrV), which is closely related to HSV‐1 and usually fatal in all animal species except for pigs. This PrV mutant, designated PrV‐ΔUL21/US3Δkin, lacks tegument protein pUL21 and carries a mutation in the active site of the pUS3 protein kinase. While the deletion of most nonessential viral genes had either no or only a slight effect on neuroinvasion, neurovirulence and survival time of infected mice [29], most mice surprisingly survived infection with PrV‐ΔUL21/US3Δkin [28]. As in human HSE, lymphohistiocytic inflammation with pronounced neuronal necrosis was predominantly confined to the temporal as well as to the frontal lobes and insular cortex. With progression of the inflammatory reaction, mice revealed behavioral abnormalities such as “star gazing,” which seem to be comparable to behavioral alterations in humans suffering from HSE. Strikingly, only few mice developed severe disease between day 10 and 13 *post infectionem* (pi), while the majority of infected animals were only moderately affected and able to survive despite extensive neuropathological changes.

In the present study, we analyzed survival as well as clinical and histopathological short‐ and long‐term consequences and compared the inflammatory reaction and associated neuropathological changes to further validate the PrV‐mouse model for human HSE.

## 2 MATERIAL AND METHODS

### 2.1 Animal experiments

All animal experiments were approved by the State Office for Agriculture, Food Safety and Fishery in Mecklenburg‐Western Pomerania (LALFF M‐V) with reference number 7221.3‐1‐064/17. ARRIVE guidelines 2.0 were followed as reported below.

In general, 6‐ to 8‐week‐old female CD‐1 mice were purchased from Charles River Laboratory and housed in groups of maximum five animals in conventional cages type II L under BSL 2 conditions at a temperature of 20–24°C. Mice were kept under a 12 h light–dark cycle (day light intensity 60%) with free access to food (ssniff Ratte/Maus – Haltung) and clean drinking water. Bedding (ssniff Spezialdiäten Abedd Espen CLASSIC), nesting (PLEXX sizzle nest), and enrichment material (PLEXX Aspen Bricks medium, mouse smart home, mouse tunnel) were provided. An acclimatization period of at least 1 week was allowed prior to inoculation. Animals were anesthetized with 200 µl of a mixture of ketamine (60 mg/kg) and xylazine (3 mg/kg) dissolved in 0.9% sodium chloride which was administered intraperitoneally. Afterwards, a total of 5 µl of PrV‐∆UL21/US3∆kin suspension in cell culture media was inoculated in each nostril (1 × 10^4^ plaque forming units [PFU]). Control mice were inoculated with cell culture supernatant from rabbit kidney (RK13) cells (minimum essential medium (MEM) + 5% fetal calf serum [FCS]) accordingly. Mice were monitored 24/7 and scored for clinical signs as described earlier [28]. The animals were sacrificed under deep anesthesia with isoflurane, cardiac bleeding, and final decapitation. In order to allow an unbiased investigation, as well as considering animal welfare conditions, treatment and time points of analysis were determined for each individual animal prior to the experiment by simple randomization. Blinding was performed during allocation of animals and data analysis. The minimum number of animals necessary in the different exploratory experiments were calculated on a disease incidence of 80% of inoculated animals and 0% in mock‐infected mice (power = 0.8, *α* = 0.1).

#### 2.1.1 Long‐term investigation

Long‐term effects were determined clinically and histopathologically over 6 months in an exploratory study. PrV‐∆UL21/US3∆kin‐infected animals (*n* = 5) each were analyzed by histology at 28, 35, 42, 49, 84, and 168 days pi. Mock‐inoculated mice (*n* = 6) were included as control.

#### 2.1.2 Neurohistopathological analyses of early inflammation

We reused mouse brain tissue sections obtained from the previous experiment [28] to explore pathomorphological changes and the spatial distribution of infiltrating immune cell populations during the first 21 days of infection in detail. Mice sacrificed at 2, 8, 12, 15, and 21 days pi (*n* = 3) served as positive material. Mock‐infected mice were included as control (*n* = 3).

#### 2.1.3 Immunological analyses of early inflammation

To assess neuroinflammatory response by flow cytometry PrV‐∆UL21/US3∆kin‐infected (*n* = 6) as well as control mice (*n* = 4) were sacrificed at 2, 8, 12, 15, and 21 days pi to identify and quantify inflammatory infiltrates and cytokine levels in the brain.

#### 2.1.4 Neurohistopathological analyses of severely diseased mice

PrV‐ΔUL21/US3Δkin‐infected animals from the different experiments (*n* = 6) that show severe clinical signs or found dead were analyzed histopathologically to explore the severe disease outcome. Animals from the first trial to determine the mean time to death and the kinetic study [28] as well as the long‐term experiment (this study) were included.

### 2.2 Virus

PrV‐ΔUL21/US3Δkin was generated in a PrV‐Kaplan (PrV‐Ka) [30] background as described previously [28]. The virus was propagated in RK13 cells grown at 37°C in MEM supplemented with 10% FCS (Invitrogen).

### 2.3 Histopathological analysis

For histopathological investigation, the skull was removed and the head was fixed in 4% neutral‐buffered formalin for at least 1 week followed by decalcification for 3 days in Formical 2000 (Decal, Tallman, NY). From all heads, eight coronal head sections were obtained, embedded in paraffin wax and cut at 3 or 5 µm thick slices, respectively, for further histological and immunohistochemical evaluation [28]. The slices were mounted on Super‐Frost‐Plus‐Slides (Carl Roth GmbH, Karlsruhe, Germany) and stained with hematoxylin–eosin for detailed neuropathological analysis of CNS inflammation.

#### 2.3.1 Special stains

Axonal density was visualized by Bielschowsky's silver impregnation. Dewaxed paraffin sections were treated with 0.25% potassium permanganate solution (3 min) and rinsed in distilled water. Afterwards, 1% potassium sulfate solution was applied to sections (1 min). Sections were rinsed in tap and distilled water before samples were probed with 2% silver nitrate solution overnight. Sections were rinsed in distilled water (3–5 s, 2 times) and incubated with 10% ammoniacal silver nitrate solution (10 min). Sections were dipped in distilled water (5 s) and reduced in 4% formalin.

Myelination was evaluated using Luxol Fast Blue‐Cresyl Violet staining. Dewaxed paraffin sections were treated with xylol (2x 2 min), 99.5% (2x 3 min), 95%, 80%, 70%, 50% 1‐propanol (3 min each), and distilled water (3 min). After incubation in isopropyl alcohol (15 min), the section were left in luxol fast blue solution (24 h, 57°C), rinsed with distilled water, and differentiated in 0.05% lithium carbonate solution (15 s) and 70% ethyl alcohol (15 s). Sections were rinsed with distilled water and counterstained with 0.1% Cresyl Fast Violet solution, dehydrated in 96% ethyl alcohol (2x 4 min), isopropanol (1x 4 min), and butyl acetate (1x 4 min), and coated with Entellan (Merck, Darmstadt, Germany).

Mineralization was investigated using the von Kossa stain. As described above dewaxed paraffin‐embedded section were rehydrated and subsequently incubated with 5% silver nitrate solution (120 min) in the dark. After washing in distilled water, the sections were treated with 1% pyrogallic acid (4 min) and 4% sodium thiosulfate (5 min). The sections were rinsed in tap water (10 min) and counterstained with nuclear fast red (5 min), washed in distilled water, and dehydrated through graded alcohol.

Hemosiderosis following hemorrhages was assessed by Prussian Blue staining. Rehydrated paraffin‐embedded tissue sections were immersed in 1% hydrochloric acid and 2% potassium ferrocyanide (30 min), rinsed in distilled water, followed by counterstain with nuclear fast red (5 min). Sections were rinsed in distilled water and dehydrated.

#### 2.3.2 Immunohistochemistry

Infiltrating immune cell populations were identified using antibodies against Iba‐1 (FUJIFILM Wako, polyclonal rabbit anti‐rat, 1:800, for monocytes and macrophages), CD3 (DAKO, polyclonal rabbit anti‐human T cell CD3 A452, 1:100, for T cells) and CD79a (DAKO, monoclonal mouse anti‐human CD79αcy CloneHM57, 1:50, for B cells). Antibodies against glia‐fibrillary‐acid‐protein (GFAP) (abcam, polyclonal rabbit anti‐bovine, 1:100) stained astrocytes. PrV infection was visualized using an in‐house‐generated rabbit polyclonal antibody against glycoprotein B [31].

Dewaxed and rehydrated paraffin‐embedded sections were treated with 3% of hydrogen peroxide (10 min, Merck, Darmstadt, Germany) to block endogenous peroxidases. To demask antigenic sites in tissue (except sections for PrV gB), sections were either treated with 10mM citrate buffer (2x 5 min, microwave, 500W, for GFAP, CD3) or 10mM Tris–EDTA buffer (10mM Tris base, 1mM EDTA solution, 15 min, microwave, 500W, for CD79a) followed by incubation in undiluted normal goat serum (30 min). Primary antibody incubation was followed by biotinylated goat anti‐rabbit IgG (1:200; Vector Laboratories, Burlingame, CA, for GFAP) or goat‐anti‐mouse IgG (1:200, Vector Laboratories, Burlingame, CA, for CD79a) and subsequent avidin–biotin–peroxidase (ABC) complex (Vector Laboratories) for 30 min at room temperature. For CD3 staining, sections were treated with Envison®+ System – HRP (DAKO). Positive antigen–antibody reaction was visualized using AEC‐substrate (DAKO, Hamburg, Germany). After rinsing with deionized water, the sections were counterstained with Mayer's hematoxylin for 10 min and mounted with Aquatex (Merck).

#### 2.3.3 Scoring of neurohistopathological changes

In order to characterize PrV‐∆UL21/US3∆kin‐induced encephalitis during the acute phase of infection [28] in more detail, the following brain regions were stained with hematoxylin and eosin and analyzed histopathologically: brainstem (BS) including medulla oblongata and pons, mesencephalon (MES), diencephalon (DI), temporal lobe (TL) including hippocampus, parietal lobe (PL), and frontal lobe (FL).

The above mentioned brain areas were analyzed for inflammatory changes according to a recently published protocol [32] with slight modifications as given in Table 1. Neuronal necrosis and spongiform changes (Table 1) were scored only in the TL which was the most affected brain region.

**TABLE 1:** Scoring of histopathological changes assessed on hematoxylin and eosin‐stained brain regions

	Score 0	Score 1	Score 2	Score 3
Inflammation				
Meninges/perivascular	Absent	1–2 cell layers	3–5 cell layers	>5 cell layers
Neuroparenchymal	Absent	1–15 cells	16–30 cells	>30 cells
Neuronal necrosis	Absent	Mild, scattered	Moderate, multifocal groups of neurons	Severe, coalescing groups of neurons
Spongiform changes	Absent	1%–30% of brain area affected	31%–60% of brain area affected	>60% of brain area affected

Note

The mean value was built from three biological replicates obtained in the kinetic trial [28] and five replicates per indicated time point obtained in the long‐term experiment (this study).

Axonal density in the white matter was scored according to a recent protocol [32]. Demyelination was evaluated as published earlier [33]. Parenchymal mineralization and hemosiderosis were determined absent or present. Scoring of all four parameters assessed in the TL during the acute phase of infection is given in Table 2.

**TABLE 2:** Scoring of temporal lobe tissue sections for axonal density, demyelination, mineralization, and hemosiderosis

	Score 0	Score 1	Score 2	Score 3
Axonal density	No reduction	1/3 loss	1/3‐2/3 loss	>2/3 loss
Demyelination	Absent	Scattered	Multifocal	Coalescing
Mineralization	Absent or present			
Hemosiderosis	Absent or present			

#### 2.3.4 Scoring of inflammatory cells (immunohistochemistry)

Temporal lobe infiltration by CD3^+^ T cells, CD79^+^ B cells, and Iba‐1^+^ microglia/macrophages was determined during the acute phase of infection and scored as illustrated in Table 3 based on a recent protocol [32] with few adaptions. Astrogliosis based on GFAP immunoreactivity was assessed as absent or present. Infiltration of cells was evaluated in 20x or 40x magnification (high power filed = HPF).

**TABLE 3:** Scoring details of CD3, CD79, Iba‐1, and GFAP signals in temporal lobe sections

	Score 0	Score 1	Score 2	Score 3
CD3				
Meningeal/perivascular (40x)	Absent	2 layers	3–5 layers	>5 layers
Neuroparenchymal (40x)		<10 cells	11–20 cells	>20 cells
CD79				
Meningeal/perivascular (20x)	Absent	1 layer	2 layers	>2 layers
Neuroparenchymal (20x)		<10 cells	11–20 cells	>20 cells
Iba‐1				
Meningeal/perivascular (40x)	Absent	2 layers	3–5 layers	>5 layers
Neuroparenchymal (40x)		<10 cells	11–20 cells	>20 cells
GFAP				
Meningeal/perivascular (20x)	Absent or present			
Neuroparenchymal (20x)				

### 2.4 Cell preparation and antibody staining for flow cytometric analysis

Brain samples were prepared for single‐cell isolation according to a recently published protocol [34] with slight modifications. Briefly, after removing from the scull, brains were immediately transferred to ice‐cold PBS and kept on ice. The cerebellum was removed and the remaining brain cut into small pieces on ice. For cell isolation, brain pieces were pressed through a cell strainer (70 µm, BD Biosciences, Heidelberg, Germany), homogenized, and taken up in 2 ml cOmplete^TM^ Mini EDTA‐free protease inhibitor cocktail (Roche, Basel, Switzerland). Half of the homogenate was used for the analysis of the infiltrating immune cells or cytokines. The homogenate was centrifuged (286 × *g*, 4 °C, 5 min), and the supernatant was discarded. The cell pellet was resuspended in 1 ml digestion buffer (Liberase with low thermolysin concentration to a concentration of 2 U/ml in Hanks Balanced Salt Solution [HBSS] containing calcium [Ca] and magnesium [Mg]) and incubated for 30 min at 37°C with gentle agitation. The suspension was pressed through a cell strainer (70 μm), washed with 10 ml of DNAse‐free washing buffer (HBSS (Ca/Mg free) containing 10% of FCS), and centrifuged (286 × *g*, 18°C, 5 min). The supernatant was discarded, and the cell pellet was carefully resuspended in 5 ml density gradient medium (25%, room temperature), and centrifuged (521 × *g*, 18°C, 30 min, acceleration/deceleration = 0). The myelin layer and the supernatant were aspirated, and the cell pellet was resuspended in 10ml DNAse‐free washing buffer and centrifuged again (286 × *g*, 10°C, 5 min). The supernatant was discarded, and the cells were resuspended in 100µl of cold washing buffer. Cell counting and assessment of cell viability were achieved using trypan blue staining (dilution 1:10).

For flow cytometric antibody staining, the cell pellet of 1 ml brain homogenate was suspended in FACS‐buffer (PBS containing 0.1% Sodium azide and 0.1% BSA) and treated with CD16/CD32 Fc‐Receptor blocking reagent (2.5 μg/ml). Cells were stained with primary antibodies listed in Table 4 for 15 min at 4°C in the dark. For staining of whole blood, erythrocytes were lysed after surface staining by conventional lysis buffer (1.55 M NH_4_Cl, 100 mM KHCO_3_, 12.7 mM Na_4_EDTA, pH 7.4, in distilled water). Gating is shown in SI 1.

**TABLE 4:** Antibodies used in flow cytometric analyses

Antigen	Host	Isotype	Conjugate	Clone	Manufacturer
B220	Rat	IgG2a	PerCP‐Cy5.5	RA3‐6B2	eBioscience
CD3	Armenian hamster	IgG1	BUV395	145‐2C11	BDBiosciences
CD4	Rat	IgG2b	BV510	GK1.5	BioLegend
CD8	Human	IgG1	PE‐Cy7	REA601	Miltenyi
CD11b	Rat	IgG2b	BV711	M1/70	BioLegend
CD45	Rat	IgG2b	FITC	30‐F11	BioLegend
Ly6G	Rat	IgG2a	AF700	1A4	BDBiosciences
NK1.1	Mouse	IgG2a	BV786	PK136	BioLegend

### 2.5 Cytokine assay

For cytokine analysis in the brain, the LegendPlex^™^ Mouse Anti‐Virus Response Panel was used to quantify 13 mouse cytokines including interferons IFN‐α, IFN‐β, and IFN‐γ; interleukins IL‐1β, IL‐6, IL‐10, and IL‐12 as well as chemokines CCL2, CCL5, CXCL1, CXCL10, TNF‐α, and GM‐CSF, according to manufacturer’s instructions (BioLegend, Koblenz, Germany).

### 2.6 Statistical analysis

Statistical analyses and graphical visualization of data were performed using Graph Pad Prism (Version 8.4.2). To analyze brain immune cell infiltration and cytokines, ordinary one‐way ANOVA with Holm‐Sidak’s post hoc test was performed to compare infected animals from 2, 8, 12, 15, and 21 days pi to all control mice. Values with *p* ≤ 0.05 were considered significant and are indicated by asterisks (*).

## 3 RESULTS

### 3.1 Long‐term dynamics and clinical signs after PrV‐∆UL21/US3∆kin infection

In our first study [28], we monitored PrV‐∆UL21/US3∆kin‐infected mice for 21 days and investigated viral spread and inflammatory reaction in a detailed kinetic experiment. The animals developed meningoencephalitis starting at day 9 pi. Interestingly, the majority of mice showed only mild‐to‐moderate clinical signs or remained completely asymptomatic despite of an extensive inflammatory reaction, which could be detected until the end of the study. The animals were able to survive, with the exception of three mice, which died or had to be euthanized between 9 and 13 days pi. Notably, localization of viral antigen, which was detectable until day 15, and the very pronounced inflammatory response localized to the temporal lobe were largely comparable to human HSE. Mice also developed behavioral alterations including star gazing which may resemble abnormalities observed in human patients. Based on these data, we aimed to investigate the further course of infection including clinical alterations as well as central nervous lesions beyond 21 days in a long‐term experiment. To this end, clinical signs of PrV‐∆UL21/US3∆kin‐infected mice were recorded until day 168 pi. For this experiment, 47 PrV‐∆UL21/US3∆kin‐infected mice and six control mice were used. Starting from day 28 pi, five infected animals and one mock‐infected mouse each were sacrificed on days 35, 42, 49, 84, and 168 pi for neurohistopathological examination.

**FIGURE 1:**
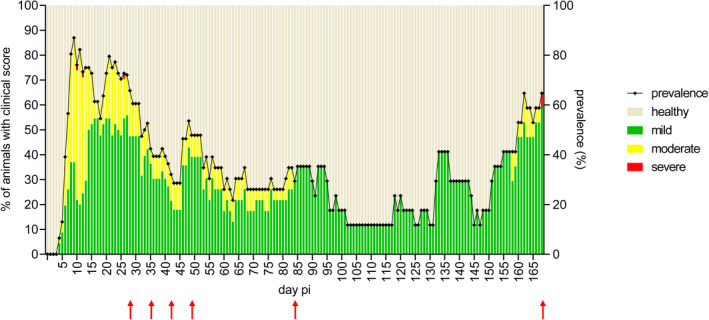
Number of diseased animals in proportion to the total number of infected animals over 168 days. The percentage of healthy (beige), mildly affected (green), moderately affected (yellow) and severely affected (red) animals is shown on the left y‐axis. The incidence at each time point is indicated by a dotted line and shown on the right y‐axis. Red arrows indicate the time points (28, 35, 42, 49, 84, and 168 days pi) at which five infected mice and one control mouse each were sacrificed and analyzed histopathologically [Colour figure can be viewed at wileyonlinelibrary.com]

As observed previously [28], at day 5 pi few mice (6%) started to show clinical signs typical for PrV‐∆UL21/US3∆kin infection. Subsequently, the incidence of clinical signs increased and reached almost 50% on day 8 pi. On day 10 pi, 87% of animal showed clinical signs which was the highest incidence detected in this experiment. On day 19, it decreased to 54%. Thereafter the number of mice showing clinical signs increased again to 77% on day 24 pi, thereafter decreasing continuously to 25% on day 46 pi. This was followed by a slight increase to 48% on day 53, followed by another decrease to ca. 30% on day 96 pi. The incidence from then on was low, at a maximum of around 24%. However, the incidence slightly increased again to around 30% from day 133 pi and decreased from day 146 pi to almost 18%. However, starting at day 153 pi, a new wave of clinical signs was noted which reached almost 77% at day 165 pi (Figure 1). In summary, we detected an essentially biphasic course of infection. The acute, first phase of the infection with two peaks at around days 10 and 20 slowly subsided about 3 weeks after the infection. The disease rate then remained at a low level, but increased again after about 6 months defined as the second phase of disease.

Early after infection, animals showed nonspecific clinical signs including ruffled fur or hunching, while two out of 47 animals remained clinically inapparent until day 28 pi. Several mice had mild pruritus and conjunctivitis and developed a nasal bridge edema. Out of 47 mice (72%), 34 animals developed alopecic skin erosions in various body regions including the head, limbs, abdomen or back, mainly occurring between day 8 and 14 pi. Some animals even developed multiple or recurring skin lesions. Typically, lesions were not hemorrhagic and healed within a week of onset. Out of 47 mice (32%), 15 animals started to show behavioral alterations including reduced activity levels and “star gazing” within the first two weeks pi while further 19 animals (40%) started to show clinical signs in the fourth week pi. Rather mild nervous signs were observed in 12 animals (25%) and included slight facial fasciculations mainly occurring between day 10 and 13 pi. Four mice showed mild ataxia mainly between day 12 and 14 pi. In total, three mice had to be euthanized because of severe convulsions and excitations at 11, 12, and 13 days pi.

Beyond 28 days pi, 29 out of the remaining 39 mice (74%) revealed recurring clinical signs mainly characterized by behavioral abnormalities, but also ruffled fur, hunched back or alopecia. Notably, not until day 159 pi, two mice (designated as mouse 1 and 2) developed seizures interrupted by phases with normal behavior or reduced activity levels. These two animals did not show any particular abnormalities in the acute phase of the infection. More specifically, mouse 1 showed first clinical signs as early as 5 days pi such as nasal bridge edema, ruffled fur, blepharospasm, and reduced activity. In the following, but only for a short time, the animal developed photophobia and signs of nervousness. Until the end of the study period, the mouse showed alternating phases of staring, nervousness, and hunching. Mouse 2 showed sickness from day 8 pi on with hunching, ruffled fur, and mild itch. From day 23 onwards, the animal had reduced activity levels which improved on day 40 pi. The animal was then clinically normal until day 156.

### 3.2 Long‐term CNS lesions after PrV‐∆UL21/US3∆kin infection

**FIGURE 2:**
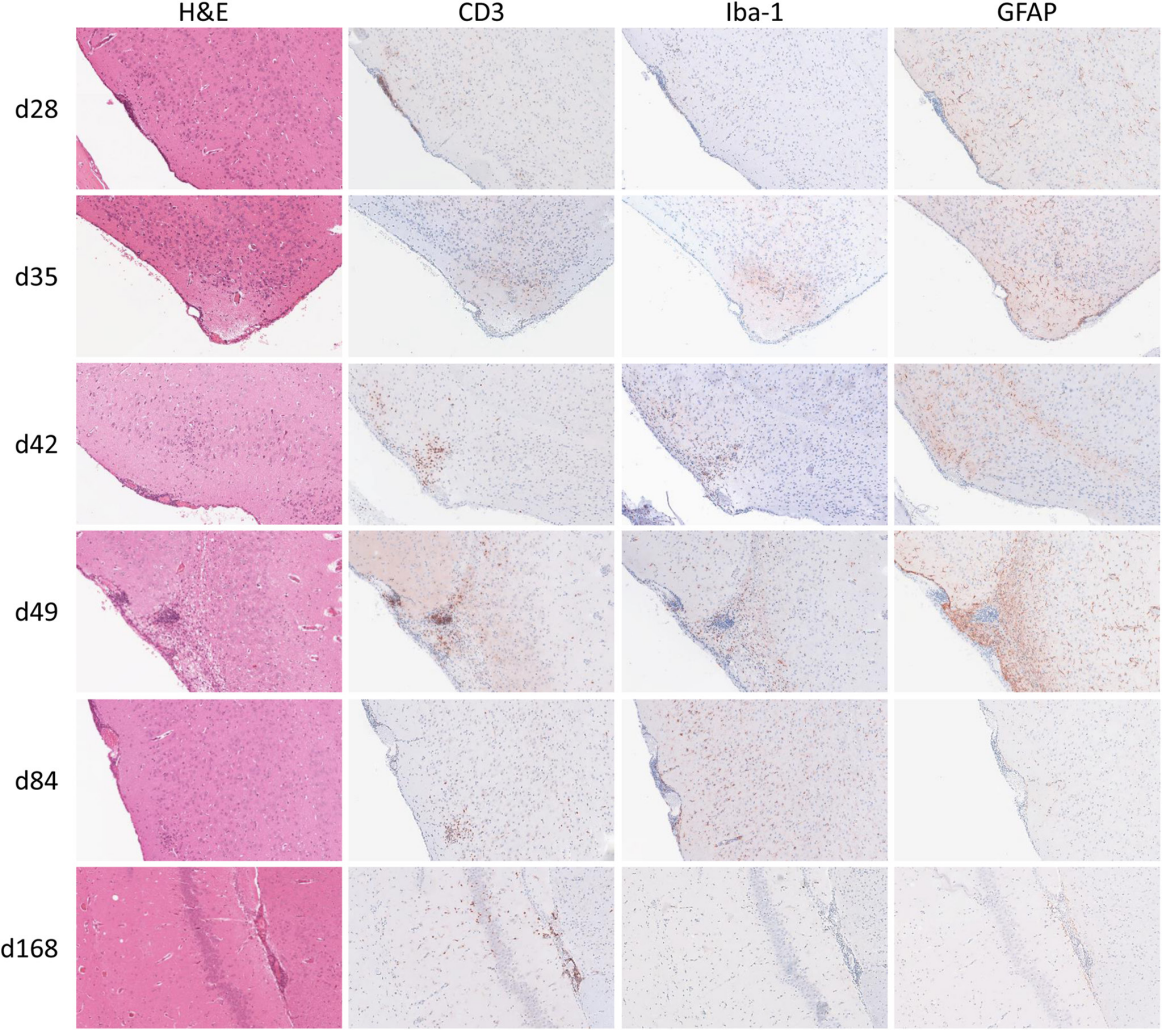
Representative images of long‐term temporal lobe lesions after PrV‐∆UL21/US3∆kin infection. At all investigated time points between day 28 and 168 pi focal mild meningoencephalitis (H&E stain) composed of CD3^+^ and Iba^+^ infiltrates (immunohistochemistry, ABC method) were detectable. GFAP^+^ glial cells (immunohistochemistry, ABC method) were generally mildly increased except in a single animal 49 days pi. Magnification 20x [Colour figure can be viewed at wileyonlinelibrary.com]

In the previous study, all mice investigated at 21 days pi showed marked meningoencephalitis [28], therefore we intended to investigate neuropathological changes also at later time points of infection. For this, five infected animals each were sacrificed at 28, 35, 42, 49, 84, and 168 days pi. The severity and location of inflammation was determined on hematoxylin and eosin stained tissue sections as described earlier [28]. Inflammatory cell infiltrates and reactive changes were differentiated by immunohistochemistry targeting CD3, Iba‐1, and GFAP on three animals each at each time point as illustrated in Figure 2.

On day 28 pi, four out of five animals revealed inflammation confined to the FL and TL. Immunohistochemistry identified mainly CD3^+^ and Iba‐1^+^ infiltrates while CD3^+^ T cells predominated in two mice. One mouse had a severe temporal meningoencephalitis with extensive necrosis of hippocampal neurons, perivascular infiltration, and mild edema. Mild astrogliosis was present in all animals. Results of hematoxylin and eosin, CD3, Iba‐1, and GFAP immunohistochemistry staining are demonstrated in Figure 2. At 35 days pi, meningoencephalitis composed of CD3^+^ and Iba‐1^+^ cells was found in all animals similar to those investigated 28 days pi. One animal showed moderate spongy changes in the BS. Likewise, all animals sacrificed on day 42 pi showed inflammatory reaction in the CNS. In one mouse multifocal gliosis was observed, while all other animals revealed mainly mild lymphohistiocytic meningoencephalitis in the TL and FL, confirmed by CD3^+^ and Iba‐1^+^ immunohistochemistry. As observed earlier, mild spongiform changes were located to the TL as well as to the BS in two out of five animals. In mice investigated on day 49 pi, mild lymphohistiocytic temporofrontal inflammation and gliosis were found in three out of five animals, which were comparable to samples of 42 days pi. Later on day 84 pi, four out five animals suffered from CNS inflammation. However, in contrast to earlier time points mild, but mixed cellular inflammatory response was found consisting of moderate numbers of lymphocytes and histiocytes, admixed with few neutrophils mainly affecting the TL and FL. At the end of the experiment, on day 168 pi, two out of five animals that were scheduled to be sacrificed that day as well as one mouse which was further euthanized because of seizures showed histopathological abnormalities including mild mixed‐cellular meningoencephalitis, as seen on day 84 pi, as well as gliosis. Severe and widespread spongiform changes, but no inflammatory reactions were present in the mouse with seizures, particularly in the TL.

Despite of long‐term CNS inflammation detected in majority of animals, immunohistochemistry against PrV gB was negative except for one animal examined 49 days pi, showing few positive neurons located in the PL and TL as well as another animal 84 days pi with a single positive neuronal signal in TL (SI 2).

### 3.3 Kinetics and distribution of the CNS inflammatory response in the acute phase

The first term of our long‐term study reflected the results of the previous experiment during the first 21 days [28]. Both indicate a biphasic course of disease with an acute phase of infection with two peaks. Mice developed meningoencephalitis from day 9. As the degree of inflammation progresses, the animals developed predominantly moderate clinical signs, including behavioral alterations, but mostly survived the infection. Only six mice died during this critical phase in three independent experiments (mean time to death and kinetic study [28] and long‐term experiment [this study]). We therefore aimed to analyze this acute and critical phase in more histopathological detail.

**FIGURE 3:**
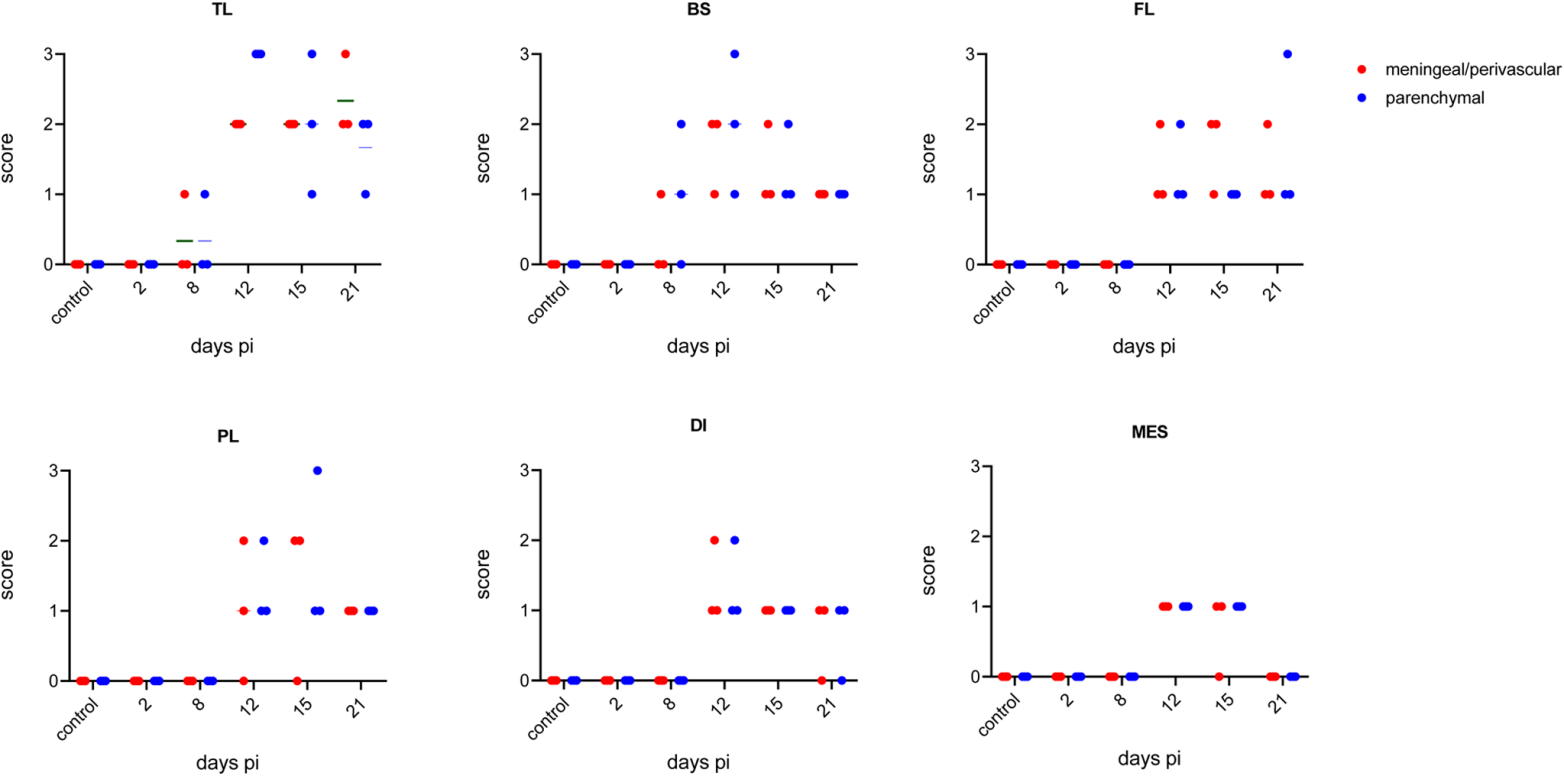
Semiquantitative scoring of meningeal/perivascular (red) and parenchymal inflammation (blue) affecting different brain regions. H&E stained sections of three mice per time point including the temporal lobe (TL), brain stem (BS), frontal lobe (FL), parietal lobe (PL), DI (diencephalon), and MES (mesencephalon) were scored [Colour figure can be viewed at wileyonlinelibrary.com]

Eight coronal head sections were stained with hematoxylin–eosin. Different parts of the brain including the BS, MES, DI, TL, PL, and FL of three animals each sacrificed at 2, 8, 12, 15, and 21 days pi were evaluated for meningeal/perivascular and parenchymal inflammatory infiltration based on a score from 0 to 3 (Table 1). Figure 3 illustrates the dynamics and score per animal and brain area at the indicated time points.

**FIGURE 4:**
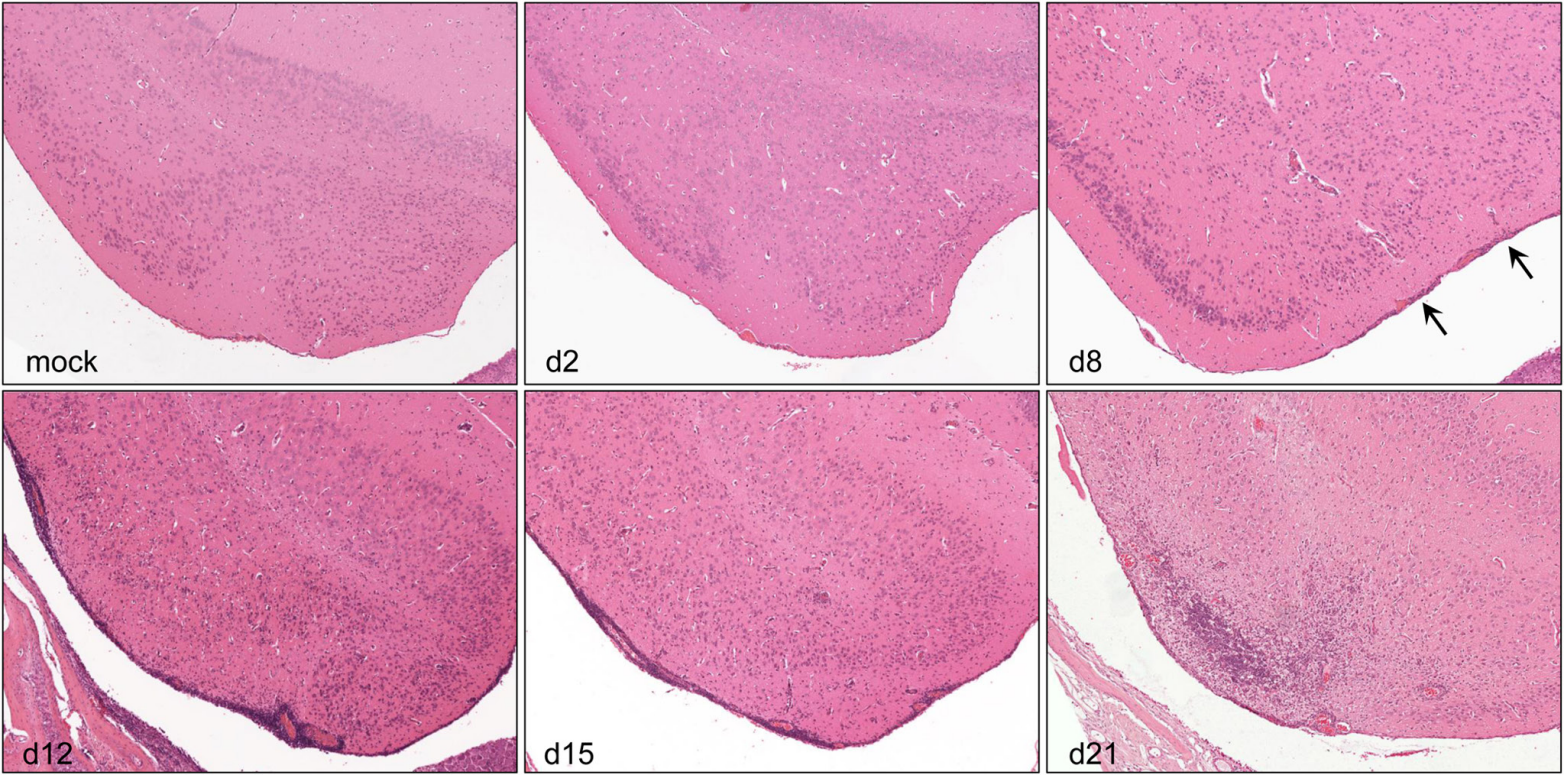
Temporal lobe sections of mice sacrificed at different early time points. Few meningeal lymphocytic infiltrates are detectable 8 days pi (arrow). Meningeal/perivascular and parenchymal infiltrates increase from day 12 to 21 pi, both resulting into marked lymphohistiocytic meningoencephalitis. Hematoxylin and eosin stain, magnification 10x [Colour figure can be viewed at wileyonlinelibrary.com]

While infected animals showed no histopathological changes at 2 days pi, meningeal/perivascular and parenchymal infiltration, respectively, affecting the BS and TL were mildly present in single PrV‐ΔUL21/US3Δkin‐infected mice sacrificed at 8 days pi. However, severe meningoencephalitis was observed in all animals sacrificed 12 days pi mainly affecting the TL. While moderate inflammation was present in BS, moderate to only mild inflammatory response was observed in DI, PL, FL, and MES. However, up to severe inflammation was found 15 days pi in the TL. Whereas at this time meningoencephalitis slightly receded in the BS and DI, inflammation moderately increased in the PL at 15 days pi. Inflammatory reaction in the FL and MES remained constantly mild to moderate. At 21 days pi, all animals still showed CNS inflammation especially in TL. Compared with the relatively mild inflammatory response in FL at 15 days pi, this brain region was more severely affected in single individuals at 21 days pi. Other brain areas such as the BS, DI, and PL showed largely reduced inflammation, while no infiltrates were detectable in the MES at 21 days pi. Thus, the TL was identified as the primary site of sustained inflammation which was first detectable at day 8 pi, peaked at 12 days pi, and remained almost consistently severe until 21 days pi (Figure 1). Representative hematoxylin and eosin‐stained sections of the TL are given in Figure 4.

The degree of neuronal degeneration and necrosis in the TL was semiquantitatively scored at the indicated time points. Although no degeneration of neurons was observed 2 and 8 days pi, loss of neurons ranged from mild to severe at 12 days pi, when inflammation increased. With ongoing inflammation 15 and 21 days pi, all animals revealed moderate neuronal degeneration (SI 3).

No hemorrhage, mineralization, or demyelination was detected in any brain section at any time point of infection.

### 3.4 Identification and spatial distribution of inflammatory effector cells

**FIGURE 5:**
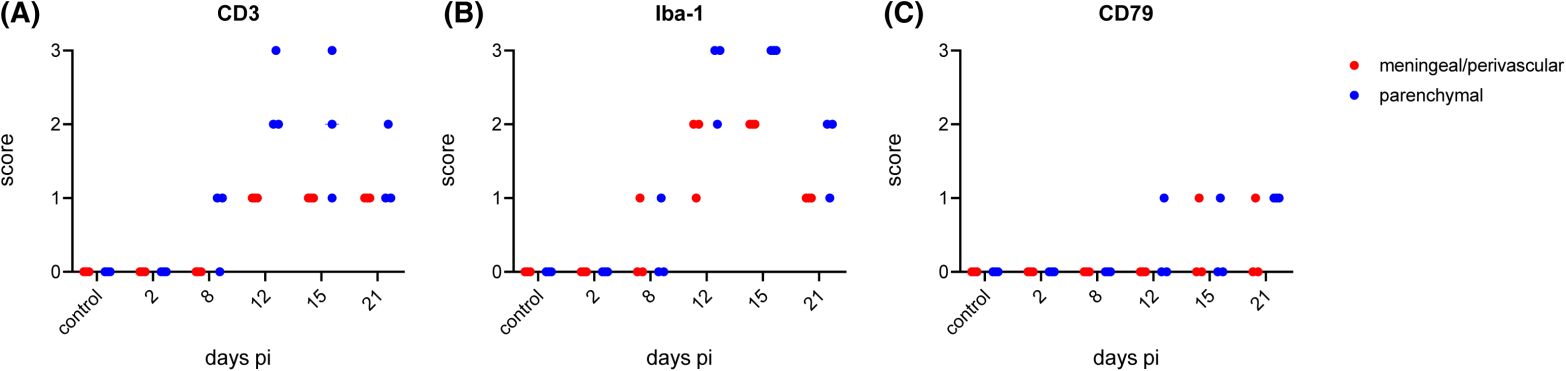
Semiquantitative scoring of meningeal/perivascular (red) and parenchymal infiltration (blue). Brain sections stained for immune cell infiltrates were scored for CD3^+^ T lymphocytes (A), Iba‐1^+^ monocytes/macrophages (B), and CD79^+^ B lymphocytes (C) in the temporal lobe (TL). Three mice each were analyzed at the indicated time points [Colour figure can be viewed at wileyonlinelibrary.com]

Tissue sections of the TL, which was most severely affected by inflammation, were further analyzed for identification of infiltrating or CNS resident immune cells, using immunohistochemistry against CD3^+^ T cells, CD79^+^ positive B cells, and Iba‐1^+^ monocytes/macrophages, respectively. Sections were semiquantitatively screened for the number of CD3, CD79, and Iba‐1^+^ meningeal/perivascular cell layers and neuroparenchymal infiltrates per HPF (Table 2). The respective scores are given in Figure 5.

**FIGURE 6:**
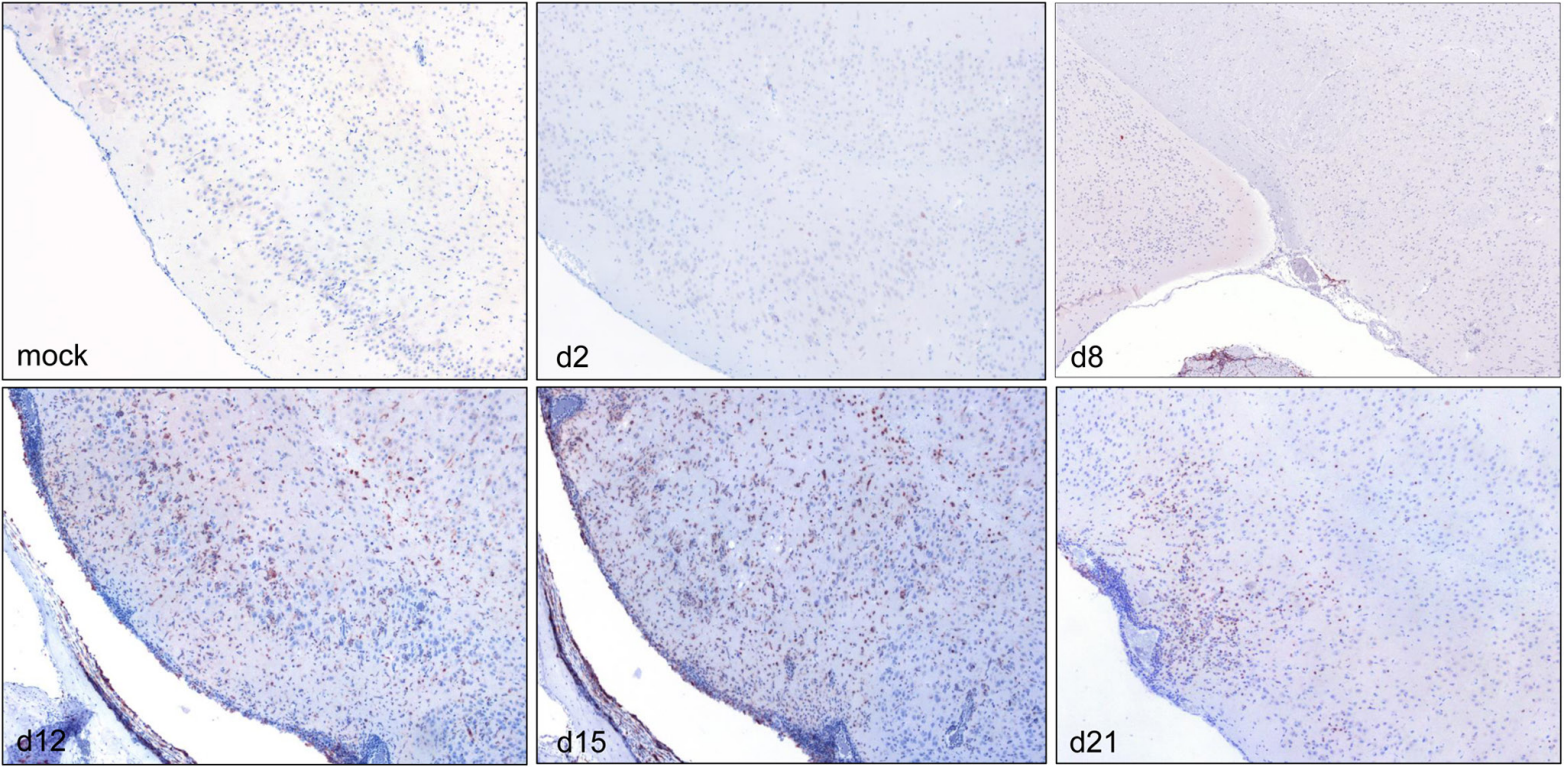
CD3^+^ T lymphocyte infiltration of the temporal lobe (TL) at different early time points pi. Representative sections of the TL showing CD3^+^ T lymphocytes at 2, 8, 12, 15, and 21 days pi. Immunohistochemistry, polyclonal rabbit anti‐human CD3^+^ antibody, ABC method, magnification 10x [Colour figure can be viewed at wileyonlinelibrary.com]

At 8 days pi, very mild meningeal infiltration of CD3^+^ T cells was detectable (Figure 5A). High numbers of CD3^+^ T cells were present 12 days pi within the parenchyma and to a lesser extent in the meninges or perivascular spaces. While meningeal and perivascular numbers of T cells remained constantly low, parenchymal infiltrating cells varied from low to high scores at 15 days pi and slightly decreased at 21 days pi. Representative tissue sections of CD3^+^ immunohistochemistry are depicted in Figure 6.

**FIGURE 7:**
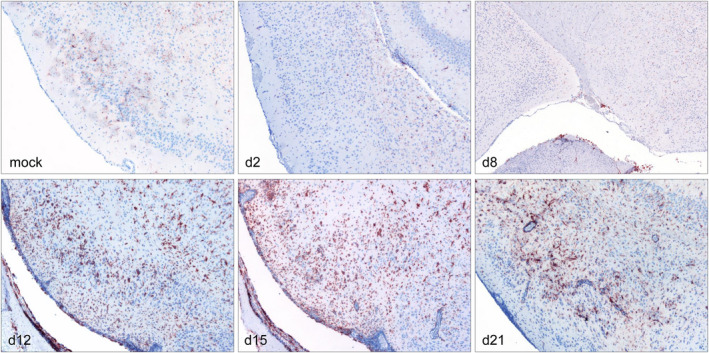
Iba‐1^+^ macrophage/microglia of the temporal lobe (TL) at different early time points pi. Representative sections of the TL at 2, 8, 12, 15, and 21 days pi showing different amounts of Iba‐1^+^ macrophages/microglia. Immunohistochemistry, polyclonal rabbit anti‐rat Iba‐1^+^ antibody, ABC method, magnification 10x [Colour figure can be viewed at wileyonlinelibrary.com]

Few Iba‐1^+^ cells were present at 8 days pi, but markedly increased within the meninges and perivascular spaces and neuroparenchyma at 12 days pi (Figure 5B). Iba‐1^+^ cells reached maximum levels in the neuroparenchyma whereas within the meninges moderate numbers were found at 12 and 15 days pi. Still considerable, but lower numbers of both parenchymal and meningeal/perivascular infiltrates were present at 21 days pi. Iba‐1^+^ immunohistochemistry stains are shown in Figure 7.

Only few CD79^+^ B lymphocytes were detectable over the study period (Figure 5C). At 12 and 15 days pi, only one mouse each showed few CD79^+^ B lymphocytes within the brain parenchyma and meninges, respectively. However, all mice sacrificed at 21 days pi revealed neuroparenchymal infiltration of CD79^+^ cells as illustrated in SI 4.

Astrogliosis was found starting 12 days pi and was present until the end of the short‐term study (SI 5).

### 3.5 Kinetics of immune cell infiltration toward the brain during the acute phase

To assay the type of infiltrating immune cells toward the CNS, brain homogenates of PrV‐∆UL21/US3∆kin‐infected mice were investigated by flow cytometry at 2, 8, 12, 15, and 21 days pi.

**FIGURE 8:**
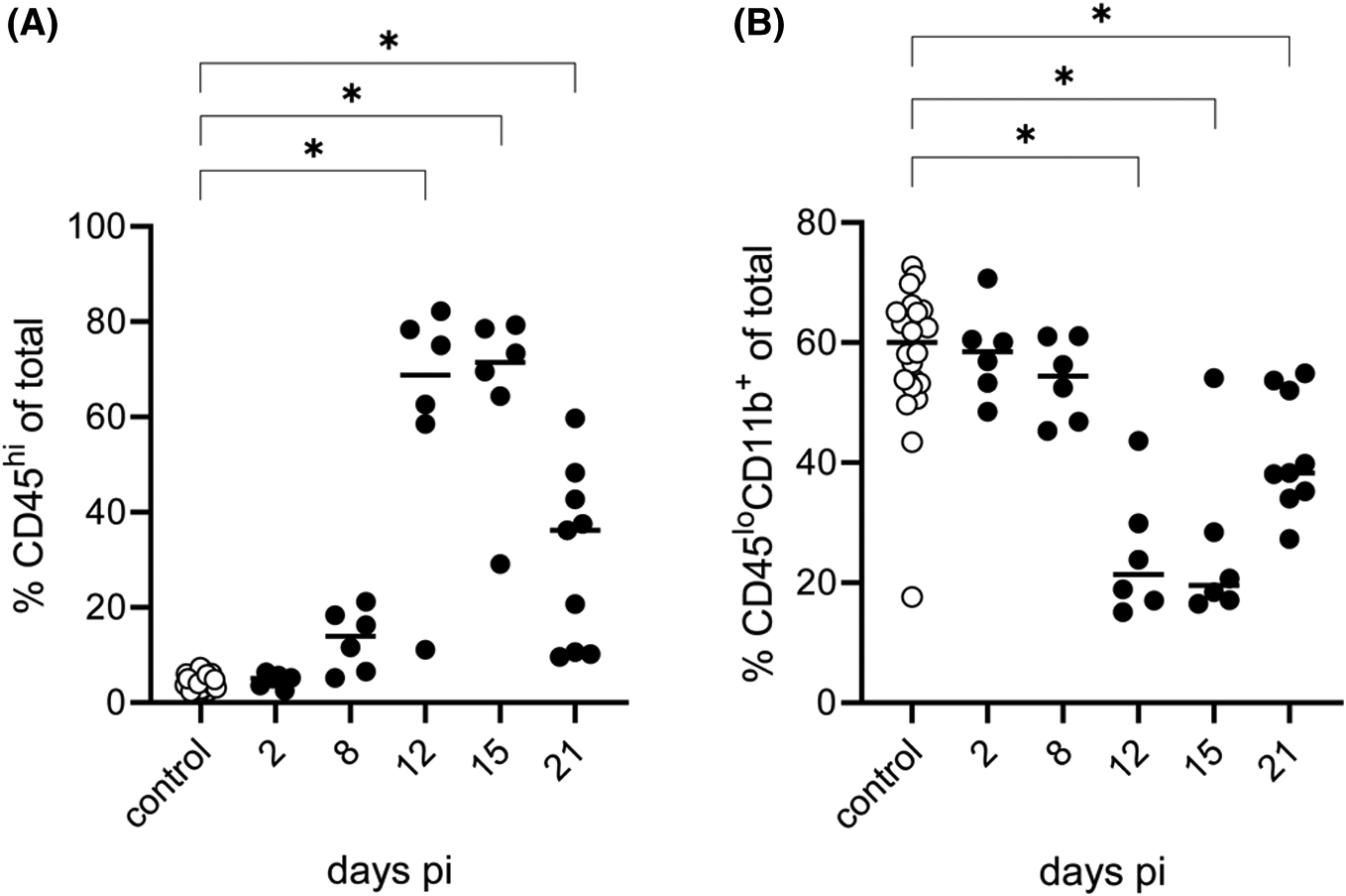
Graph over study time of CD45^hi^ brain leukocytes (A) and CD45^lo^CD11b^+^ putative microglia (B) in brain among all isolated cells from infected and mock mice. After isolation of leukocytes from murine brains via density gradient centrifugation, cells were gated for single cells and subsequently distinguished using FSC‐A and SSC‐A. Discrimination of infiltrating (CD45^hi^) and resident (CD45^lo^CD11b^+^) leukocytes was made based on CD45 and CD11b expression. Significant differences between day 0 (control mice) and infected mice at days 2, 8, 12, 15, and 21 are indicated with an asterisk (*)

**FIGURE 9:**
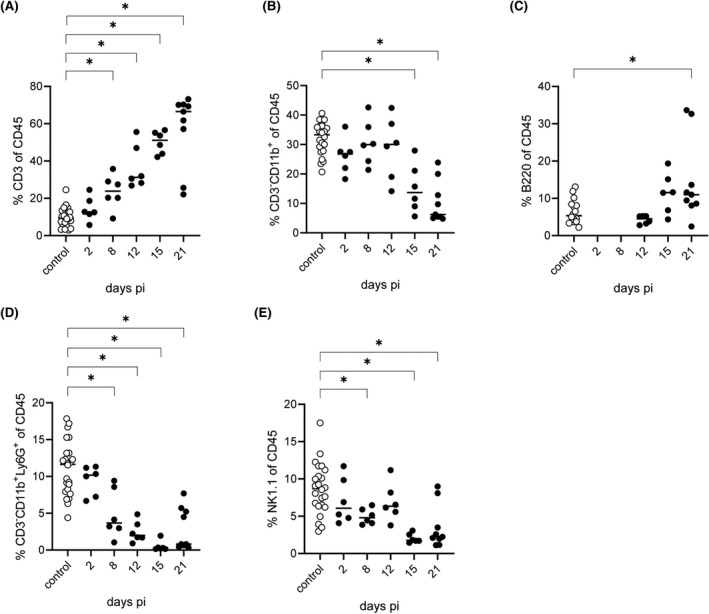
Frequencies of brain‐infiltrating CD45^hi^ leukocyte populations. Frequencies of total T cells (A) and monocytes/macrophages (B) of CD45^hi^ leukocytes were determined with an anti‐mouse CD3 or anti‐mouse CD11b antibody, respectively. An anti‐mouse B220 antibody was used to determine frequency of infiltrating B cells (C). Infiltrating granulocytes (D) were determined based on simultaneous expression of CD11b and Ly6G. Infiltrating NK cells (E) were identified by an anti‐mouse NK1.1 antibody. Significant differences between day 0 (control mice) and infected mice at days 2, 8, 12, 15, and 21 are indicated with an asterisk (*)

As shown in Figure 8A infiltrating leukocytes, defined as CD45^hi^ cells, were detectable starting 8 days pi, reaching average frequencies of up to 70% 12 and 15 days pi and declining at the end of the study (21 days pi) to 40%. In contrast, the resident leukocyte population, putative microglia defined as CD45^lo^CD11b^+^ cells (Figure 8B), was proportionally higher in control animals as well as in infected animals investigated on day 2 pi with about 60% of total cells. Nevertheless, from day 8 onwards, the fraction decreases in favor of the infiltrating CD45^hi^ leukocytes. In uninfected animals, CD45^hi^ cells consisted of approximately 10% T cells, 30% CD3^−^CD11b^+^ monocyte/macrophages (Figure 9A,B), 5% B cells (Figure 9C), and 10%–15% granulocytes (Figure 13D). Natural killer (NK) cell frequency was highly variable (Figure 9E). After infection, higher numbers of CD3^+^ T cells were found starting at 8 days pi (Figure 13A). Whereas at day 12 pi, CD3^+^ T cell frequencies were comparable to CD3^−^CD11b^+^ monocytes/macrophages, T cells markedly increased 15 days pi at the expense of monocytes/macrophages (Figure 9A,B). The number of T cells reached almost 70% at 21 days pi, whereas CD3^−^CD11b^+^ monocytes/macrophages decreased by two‐thirds in total until 21 days pi. In addition, B cells were found 15 days pi reaching frequencies of around 10% until the end of the study. The number of granulocytes was highest on day 2 pi with about 10% and then decreased continuously until 21 days (Figure 9D). The population of NK cells only accounted for a small proportion of the inflammatory infiltrate, and their frequency ranged around 5% at 2, 8, and 12 days pi while it decreased at later time points (Figure 9E).

**FIGURE 10:**
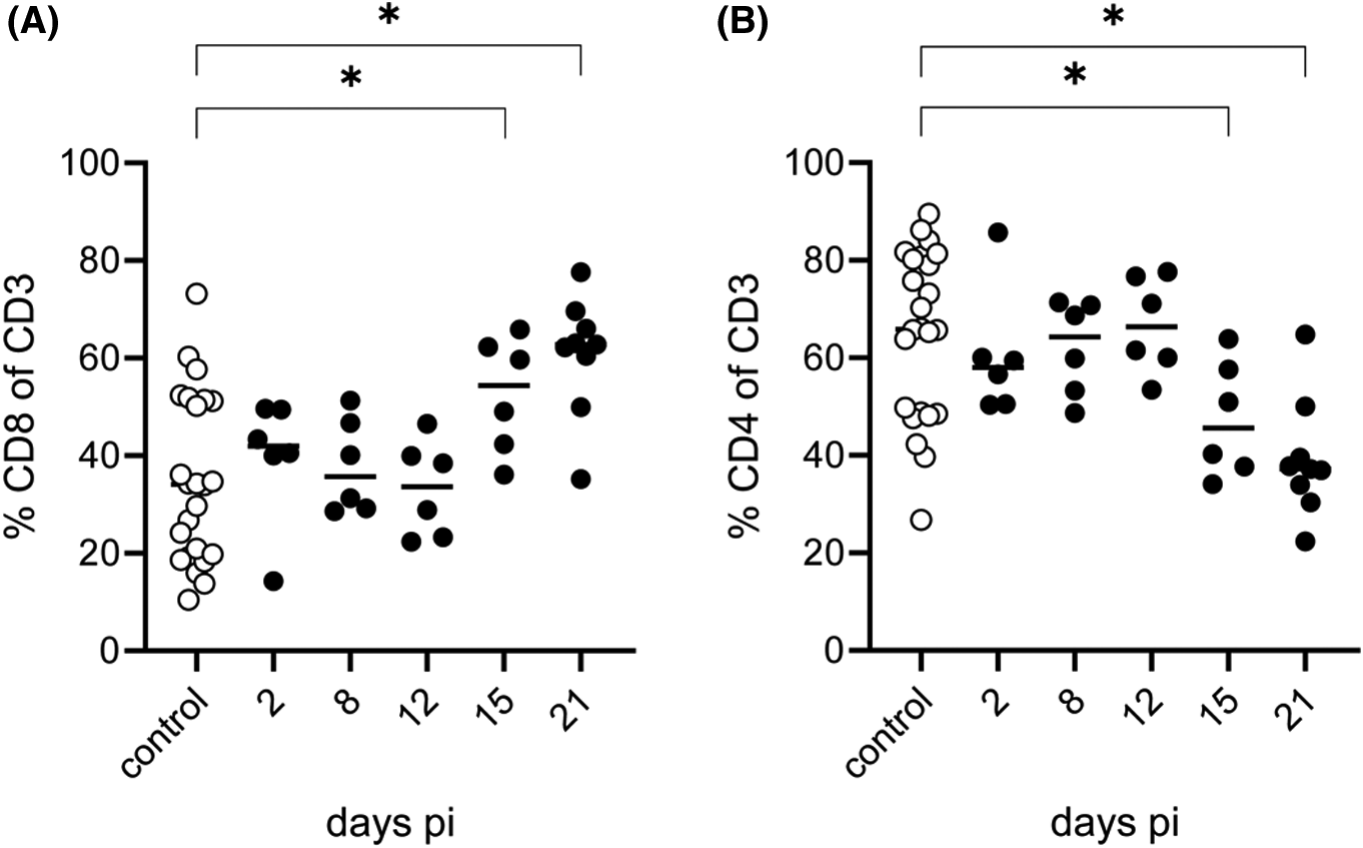
Summary graphs of T cell subpopulations among infiltrating leukocytes. Frequency of total T cells of CD45^hi^ leukocytes was determined with anti‐mouse CD3 antibody and quantified over 21 days pi. Further differentiation into cytolytic and helper T cells was based on the expression of either CD8 (A) or CD4 (B), respectively. Significant differences between day 0 (control mice) and infected mice at days 2, 8, 12, 15, and 21 are indicated with an asterisk (*)

To further characterize T cell infiltration, CD4^+^ and CD8^+^ subpopulations of T cells were determined (Figure 10). As the population of cytotoxic CD8^+^ T cells was rather stable over the first 12 days with approximately 40% (Figure 10A), the frequency of CD4^+^ helper T cells (Figure 10B) was already higher at 8 and 12 days pi reaching 60%. However, at day 15 pi the ratio of CD4^+^ to CD8^+^ T cells was reversed, and the number of cytotoxic T cells increased and remained high until the end of the study reaching in average 60% while CD4^+^ T cells decreased to 40%.

**FIGURE 11:**
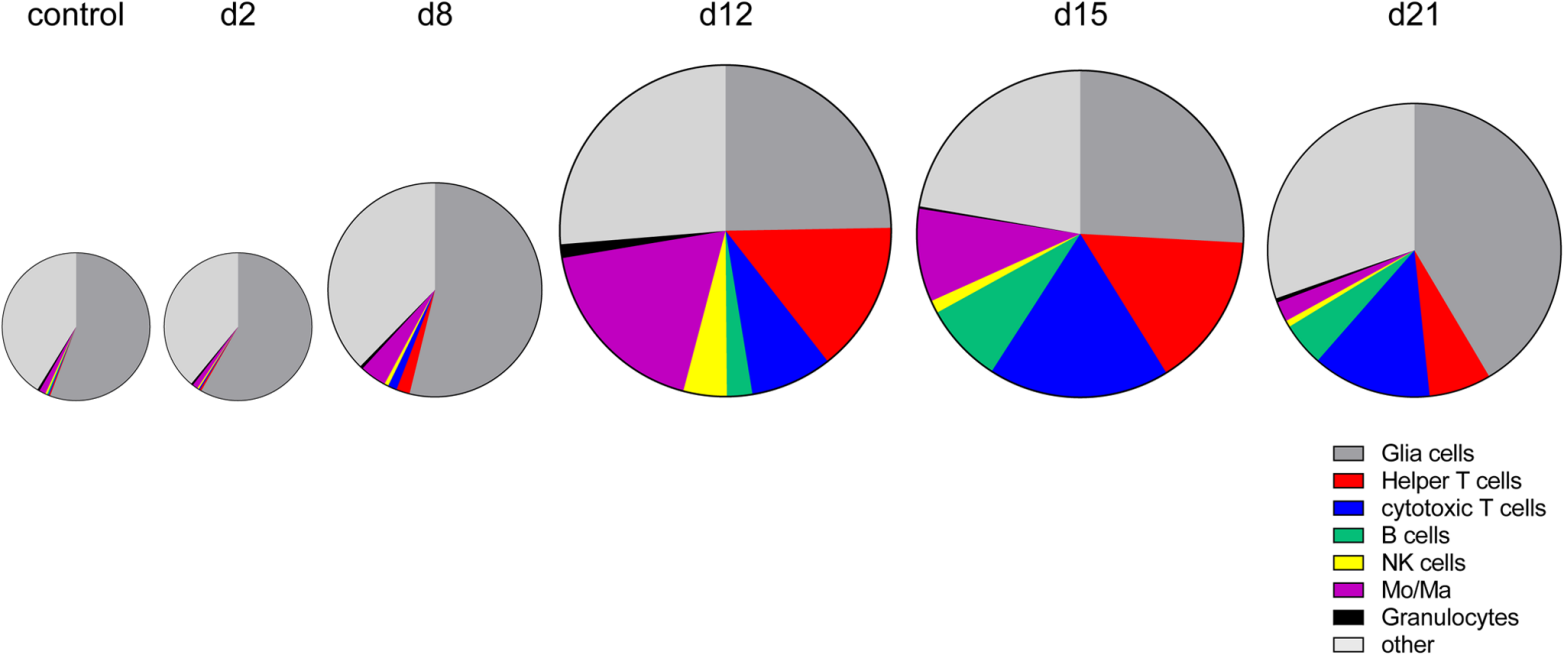
Summary presentation of infiltrating and resident cell populations based on the total cell count over the study period. Sizes of pie charts indicate the number of infiltrates and severity of inflammation on the different time points. Brain resident glial cells as part of the total cell population serve as a reference point for the infiltrating cell populations [Colour figure can be viewed at wileyonlinelibrary.com]

In summary, leukocyte infiltration toward the brain was detectable starting on day 8 pi (Figure 11). As measured based on the total cell count, the proportion of monocytes/macrophages increased up to day 12 and reached almost identical values compared with infiltrating T cells. From day 15 pi, the number of T cells markedly increased whereas monocytes and macrophages declined. Compared with day 12 and 15 pi, the overall number of infiltrating cells slightly decreased on day 21 pi.

#### 3.5.1 Kinetics of chemokine and cytokine expression during encephalitis

**FIGURE 12:**
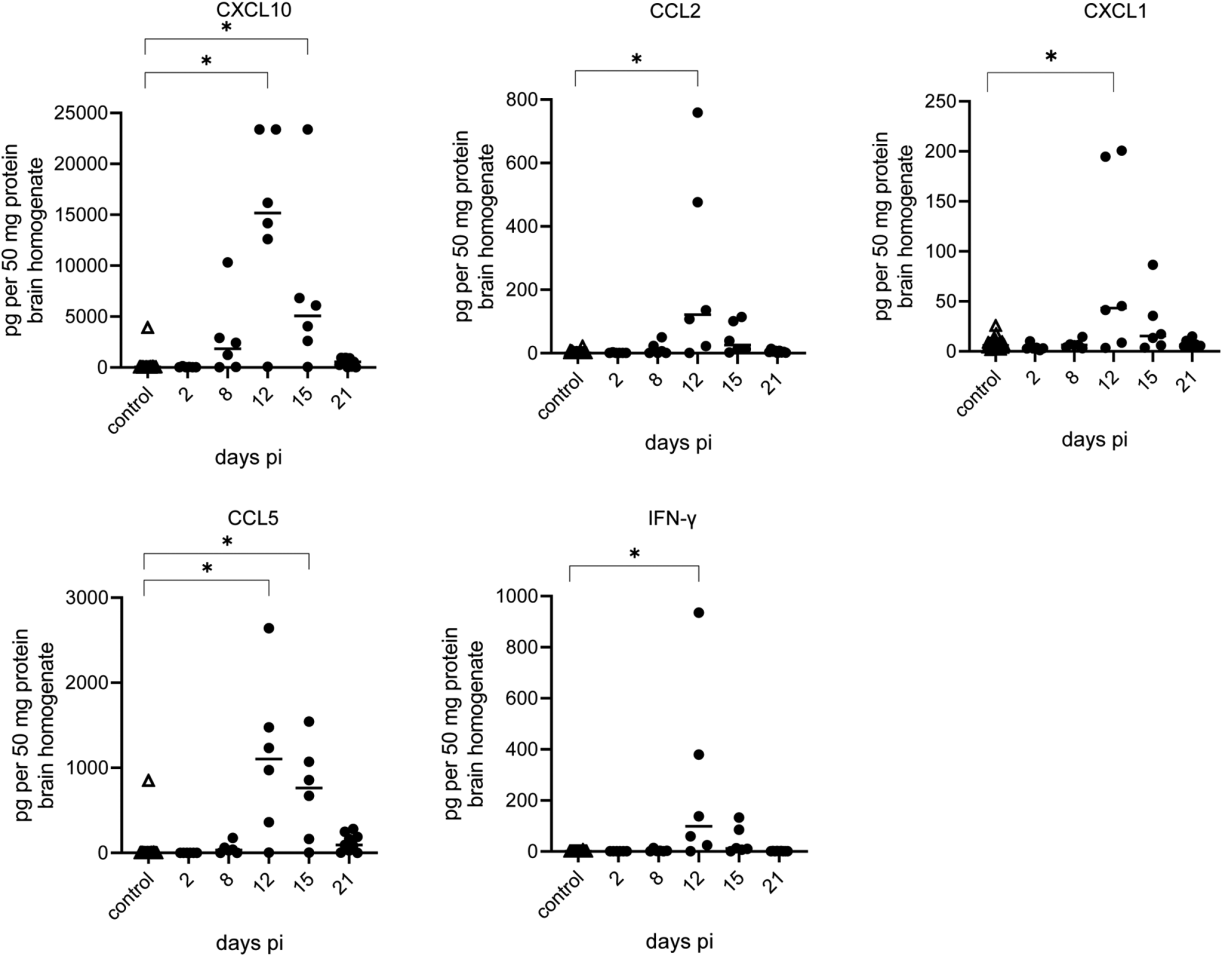
Quantification of different chemo‐ and cytokines in brain homogenate. Supernatant of brain homogenates was subjected to protein quantification and subsequently analyzed by flow cytometry. Cytokine concentrations of infected mice were measured with a commercial multiplex assay and compared with mock control mice. Significant differences between control mice and infected mice at days 2, 8, 12, 15, and 21 are indicated with an asterisk (*)

Chemokine and cytokine expression in brain homogenate was investigated by flow cytometry using a commercial LegendPlex™ Mouse Anti‐Virus Response Panel (BioLegend, Germany). Analysis revealed five cytokines and chemokines including CXCL10, CCL2, CXCL1, CCL5, and IFN‐γ to be significantly elevated in PrV‐∆UL21/US3∆kin‐infected animals (Figure 12) at 12 days pi. Levels were slightly higher at 15 day pi, but reached base line 21 days pi. Cytokines IFN‐α, IFN‐β, interleukins IL‐1β, IL‐6, IL‐10, and IL‐12 as well as TNF‐α and GM‐CSF were not elevated at the investigated time points.

### 3.6 Neurohistopathology of severely diseased PrV‐∆UL21/US3∆kin‐infected animals

From all experiments (mean time to death and kinetic study [28] and long‐term trial [this study]), seven animals in total presented with severe clinical condition and seizures, and were either euthanized (*n* = 6) or found dead (*n* = 1). In the first trial to determine the mean time to death, one mouse was sacrificed around 10 days pi, whereas two animals were euthanized or found dead at 10 and 13 days pi in the kinetic study as reported earlier [28]. Four mice were euthanized during the long‐term experiment described in the present study at days 11, 12, 14, and 168 pi because of seizures and excitations, while one animal was euthanized because of marked dermatitis at day 27 pi. This animal was excluded therefore from the following histopathological examination. In order to better understand this rare but severe clinical course which mimicked that observed in HSV‐1‐infected human patients, we analyzed the brains of these mice more closely. Brain sections of all animals were investigated using hematoxylin and eosin staining as well as immunohistochemistry for PrV antigen detection and identification of immune cells.

**FIGURE 13:**
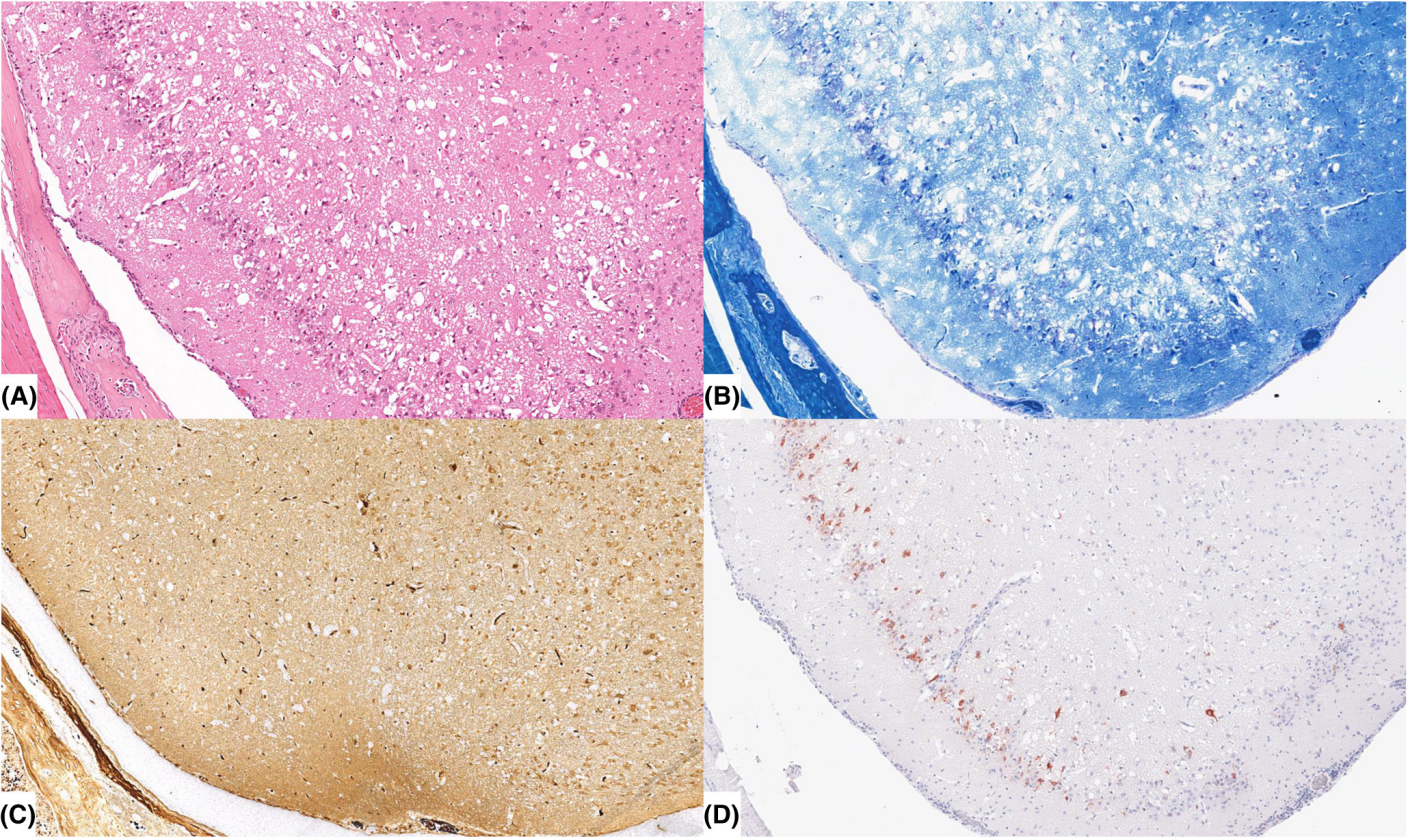
Lesions in the temporal lobe (TL) of severely diseased mice infected with PrV‐∆UL21/US3∆kin. (A) Temporal lobe (TL) of a mouse euthanized at day 10 pi because of severe general condition and seizures showing extensive spongiform changes, accompanied by marked neuronal necrosis. There is only very mild lymphohistiocytic meningoencephalitis. Hematoxylin and eosin, magnification 10x. (B) Temporal lobe (TL) sequential section of (A) with diffuse loss of myelin, Luxol Fast Blue stain, magnification 10x. (C) Temporal lobe (TL) sequential section of (A) with marked decrease of axonal fibers, Bielschowsky silver stain, magnification 10x. (D) Temporal lobe (TL) sequential section of (A) revealing abundant neurons with positive PrV glycoprotein B (gB) antigen signals, immunohistochemistry, polyclonal anti‐rabbit antibody, ABC method, AEC chromogen (red‐brown), Mayer's hematoxylin counter stain (blue), magnification 10x [Colour figure can be viewed at wileyonlinelibrary.com]

Histopathologically, in all animals sacrificed or found dead between days 10 and 14 pi, severe intra‐ and perilesional spongy changes, defined as intra‐ and extracellular edema of variable severity, mainly confined to TL, were diagnosed (Figure 13A). Extensive neuronal degeneration and necrosis but rather mild lymphohistiocytic meningoencephalitis was present which is contrast to clinically less affected mice showing rather severe inflammation. Variable loss of axons and myelin was present (Figure 13B,C). Astrocytosis was only sparsely detectable. In all six animals analyzed between days 10 and 14 pi, PrV anti‐gB immunohistochemistry revealed abundant infected neurons mainly in the TL, but also in the PL and FL (Figure 13D). Notably, in an animal sacrificed 168 days pi (end of the long‐term experiment) widespread edema, especially in the FL and TL as well as in the DI, without any inflammatory reaction was found. Details of histopathological investigation are summarized in Table 5.

**TABLE 5:** Histopathology of PrV‐∆UL21/US3∆kin‐infected mice sacrificed moribund or found dead during different experiments

Mouse	Trial	Time points of euthanasia/death	Brain area	PrV gB antigen detection	Spongiform changes	meningoencephalitis	Myelin loss	Axonal loss
1	MTD [28]	10 days	FL	–	0	0	0	0
			TL	3	3	2	2	2
			PL	3	1	1	1	1
			DI	–	0	1	0	0
			MES	–	0	1	0	0
			BS	–	0	1	0	0
2	KIN [28]	10 days	FL	1	0	0	0	0
			TL	3	1	1	0	0
			PL	3	3	1	2	1
			DI	–	0	0	0	0
			MES	–	0	1	0	0
			BS	–	0	1	0	0
3	KIN [28]	13 days^†^	FL	–	1	1	1	0
			TL	2	2	1	2	1
			PL	1	2	1	3	1
			DI	–	1	0	0	0
			MES	–	0	1	0	0
			BS	–	0	1	0	0
4	LT	11 days	FL	3	2	1	?	1
			TL	3	3	1	3	3
			PL	3	1	1	3	3
			DI	–	0	0	0	0
			MES	–	0	0	0	0
			BS	–	0	0	0	0
5	LT	12 days	FL	–	0	1	0	0
			TL	2	3	2	2	2
			PL	–	1	1	1	0
			DI	–	0	0	0	0
			MES	–	0	1	0	0
			BS	–	0	1	1	1
6	LT	14 days	FL	–	0	1	0	0
			TL	2	2	1	3	2
			PL	2	1	1	0	0
			DI	–	0	1	0	0
			MES	–	0	1	0	0
			BS	–	0	1	0	0
7	LT	168 days	FL	–	3	0	3	3
			TL	–	3	0	3	3
			PL	–	2	0	3	3
			DI	–	3	0	3	3
			MES	–	0	0	0	0
			BS	–	0	0	0	0

Note

Anti PrV‐gB immunohistochemistry as well as severity of spongiform changes, meningoencephalitis, myelin and axonal loss were determined based on a 0–3 scale.

Abbreviations: BS, brain stem; DI, diencephalon; FL, frontal lobe; gB, glycoprotein B; KIN, kinetic experiment; LT, long‐term experiment; MES, mesencephalon; MTD, mean time to death experiment; PL, parietal lobe; TL, temporal lobe.

† Animal found dead.

## 4 DISCUSSION

In this study, we further characterized our mouse model which more accurately reflects human herpesviral encephalitis [28]. Mice intranasally infected with a PrV mutant lacking the tegument protein pUL21 and kinase function of pUS3 develop disease with striking analogies to human HSE including temporofrontal lobe associated inflammation and concomitant behavioral alterations. Despite extensive meningoencephalitis the majority of animals survive, which prompted us to further investigate the dynamics of the inflammatory response and CNS lesions in a long‐term trial until 6 month after infection (168 days pi).

We demonstrate that infection with PrV‐∆UL21/US3∆kin resulted in an essentially biphasic disease pattern with slight multi‐wave dynamics. Compared with our first study, clinical data obtained during the first 21 days in this study were highly reproducible [28]. In the first, acute phase within the first week of infection, most of the animals developed clinical signs, which led to the first peak on day 9. While mice started to recover in the second week, the number of diseased animals increased again by the end of the third week leading to a second peak at day 21. At around day 50 pi, a third increase of clinical signs was detected, followed by a fourth mild increase after 4 to 5 months (130 days pi). A fifth rapid increase of disease was detected close to the end of the experiment at 168 days pi, which is regarded as the second phase of the biphasic course.

Long‐term PrV‐∆UL21/US3∆kin‐infected mice showed lymphohistiocytic inflammation at days 28, 35, 42, and 49 pi, while lymphocytes and macrophages admixed with low numbers of scattered neutrophilic infiltrates were detected in mice at days 84 and 168 pi. Neutrophils are usually present at very early time points of infection when viral antigen is detectable [35]. However, viral antigen could be shown in single animals at 49 and 84 days pi which may lead to recurrent encephalitis as reported in humans [10, 12, 35]. Moreover, this data point to the establishment of reactivation from latency in this PrV animal model because viral antigen was absent at all other time points investigated. However, this requires further investigation. In addition to possible reactivation, chronic CNS inflammation, which has been reported in mouse models [36], but rarely in humans [37, 38], should be considered based on the clinical and histopathological findings. Autoimmune encephalitis, which occurs relatively frequently after HSE [39], should also be part of further investigations. In this context, seizures which appeared approximately 6 months after PrV‐∆UL21/US3∆kin infection may result from either chronic or autoimmune encephalitis. Epilepsy has been described in chronic herpes encephalitis, but autoimmunity may also play an important role in the development of seizures [40, 41, 42].

Behavioral alterations such as star gazing and alternating phases of reduced and normal activity in PrV‐∆UL21/US3∆kin‐infected mice have not been described in any of the animal models for HSE so far. Interestingly, the majority of PrV‐∆UL21/US3∆kin‐infected animals reproducibly developed behavioral impairment, 32% in the first 2 weeks after infection, and further 40% 3 weeks after infection. In 74% of recovered mice, clinical disease returned which became obvious by behavioral changes and to a lesser extent by non‐specific clinical signs recurring beyond 28 days pi. In human HSE patients, similar long‐term symptoms are frequently reported such as memory impairment, personality and behavioral abnormalities, and epilepsy [43]. In line with our findings, mice infected with HSV‐1 strain 17 syn + exhibited severe spatial memory deficits in a long‐term trial associated with axonal degeneration and secondary demyelination in affected brain regions, and later cortical atrophy with still moderate lymphohistiocytic inflammation at day 30 and 60 pi [36].

Based on the present data, we showed that PrV‐∆UL21/US3∆kin infection leads to a biphasic course of infection. In order to better understand the inflammatory dynamics in the acute phase of infection (first 21 days), we performed detailed histopathomorphological studies. Based on the data obtained from the previous trial [28,] we decided for five key time points at day 2, 8, 12, 15, and 21 pi for the in‐depth investigation.

In general, meningoencephalitis was confined to the temporal and later also to the frontal lobe, consisting of varying numbers of meningeal/perivascular and neuroparenchymal inflammatory cell infiltrates as well as neuronal necrosis. Although no inflammatory changes were detectable 2 days after infection, the number of brain inflammatory cells consisting of differing proportions of CD3^+^ T lymphocytes, Iba‐1^+^ macrophages, and later CD79^+^ B lymphocytes were detectable at day 8 pi, peaked between day 12 and 15 pi and showed slightly lower levels at 21 days pi.

Flow cytometric analysis confirmed the dynamics and kinetics of the infiltrating immune cells, revealing that the cells in the brain were composed of up to 70% of immune cells which was also observed in an animal model for HSE [44]. At 12‐ and 15‐days pi, the concentration of CCL2 in brain suspension was elevated contributing to increased permeability of the blood brain barrier, thus enabling increased influx of immune cells including monocytes [45]. Detailed characterization of the infiltrate showed the classic course of an antiviral immune response. Although the infiltrate until day 12 was still largely composed of myeloid cells, the ratio shifted toward lymphocytes during later times. Subsequently, T cells invaded the CNS, which is further promoted by the release of CCL5, a chemokine known to specifically attract T cells [46]. Increased expression of CXCR3 on NK, CD4^+^, and CD8^+^ cells and the following interaction with increased levels of CXCL10, which is derived from various cell types including monocytes, promote a robust immune response, which was shown to prevent mortality in a murine HSV‐1 infection model of HSE [47]. Elevated levels of CCL2, CCL3, CCL5, and CXCL8 were also found in the CSF of HSV‐1‐infected humans [48]. Looking at the composition of the infiltrate at later time points (from day 12), as well as the chemokine and cytokine levels, it is striking that infection with PrV‐∆UL21/US3∆kin led to a polarization of the brain infiltrate from mainly CD4^+^ T cell response toward a Th1 chemokine profile (CXCL10) and a subsequent CD8^+^ T cell infiltration. This matches well with the two clinical peaks at 10 and 20 days pi observed in the acute phase of the disease during the first 21 days. To what extend this proinflammatory response contributes to the rarely observed fatal pathology in mice remains to be elucidated, but as all measured cyto‐ and chemokines returned to baseline levels at the end of the study, it seems that this polarization rather prevents fatal outcome by enhanced viral clearance. However, regarding the long‐term experiment and the presence of CD3^+^ T cells in the temporal lobe until 6 months after infection, the immune status of these T cells should be discussed and tested in future studies. On the one hand, prolonged persistence of CD4^+^ and CD8^+^ T cells is described after acute HSV‐1 infection, which successfully inhibits viral reactivation [49]. On the other hand, these cells may consist of exhausted CD8^+^ T cells, which have lost their functionality because of chronic or persistent infection, leading to recurring clinical disease at later time points [50].

We also further characterized the primary involvement of the TL in necrotizing, lymphohistiocytic herpetic encephalitis as it occurs in human HSE [35, 51, 52]. Primary involvement of TL in human HSE has been shown by detailed histopathological investigation of human brains obtained from patients at different time points of infection giving the time of onset of clinical signs [18]. In human patients died within the first week of onset of disease, relatively mild inflammation was present, whereas in the second and third week, inflammation was more severe, mainly affecting the meninges and cortex of TL. If this is compared with our initial kinetic trial [28] and our present investigations, the results are largely congruent, at least as judged by appearance after onset of clinical signs.

Compared with HSE mouse models, only few studies report on inflammatory lesions in the CNS in detail. After infection with HSV‐1 strain, 17 syn + few foci of necrosis and mild neutrophilic to lymphocytic inflammation were present in the trigeminal tract of mice during the first week after inoculation [36] as found in PrV‐∆UL21/US3∆kin‐infected mice [28], but also in the olfactory bulb. Half of the mice infected with HSV‐1 syn + succumbed to death between day 7 and 10 pi, showing multifocal necrosis of the piriform, entorhinal, occipital cortices, thalamus, and cerebellum and lymphohistocytic inflammation, while other animals survived [36]. Although in our model, the critical phase occurs from day 9 to 14 pi, only few PrV‐∆UL21/US3∆kin‐infected animals were seriously affected while the majority of mice survived [28] (and this study).

Within this critical phase, six mice out of a total of 122 animals used in different approaches had to be euthanized because of seizures and general bad condition. These mice differed histopathologically from all other animals, especially by extensive viral replication, concomitant with massive intra‐ and perilesional spongiform changes consistent with widespread intracellular and extracellular edema, and neuronal necrosis primarily found in TL. However, these animals showed less inflammatory reaction to infection. In HSE patients two different forms of edema have been reported, extracellular, vasogenic edema, and intracellular, cytotoxic edema [53]. Patients suffering from cytotoxic edema were generally in worse condition compared with those with vasogenic edema, which mirrors our findings. PrV‐∆UL21/US3∆kin‐infected animals with severe edema further showed extensive demyelination and loss of axons mainly in the TL. It has been proposed that increased tissue pressure caused by edema may lead to demyelination [54]. Demyelination linked to alphaherpesviral infection has been reported in HSV‐1‐infected cotton rats [55] and several mouse strains [56, 57, 58], and has even been associated with multiple sclerosis in humans [59, 60, 61]. However the functional role of herpesviruses in demyelinating diseases is still unclear, which urges the need of further research [62].

Severe forms of HSE occur only sporadically, although approx. 67% of the world's population aged between 0 and 49 years were estimated in 2016 to be infected with HSV‐1 [63]. However, subclinical, milder forms of HSE are possibly underdiagnosed [64]. Mice intranasally infected with PrV‐∆UL21/US3∆kin generally show mild‐to‐moderate clinical signs or even remain asymptomatic, while only few animals show fatal disease progression. Animals that survive the critical period either recover completely or experience recurrent disease, which may indicate reactivation from latent infection, chronic inflammation, or an autoimmune reaction toward PrV‐∆UL21/US3∆kin infection. As suggested earlier [65], an ideal animal model for herpesviral encephalitis should include (i) infection via the mucocutaneous route, (ii) a small proportion of animals showing severe disease, and (iii) a large proportion of individuals developing an immune response that protects from severe disease. In summary, although further investigations are still needed, our present findings strongly support that the PrV‐∆UL21/US3∆kin mouse is well suited to investigate the mechanisms involved in alphaherpesviral infection of the nervous system and the consequences in humans. As a long‐term goal, this animal model might guide research for an effective HSE therapy.

## CONFLICT OF INTEREST

The authors have no conflicts of interest to declare that are relevant to the content of this article.

## AUTHOR CONTRIBUTIONS

Julia Sehl‐Ewert, Ulrike Blohm, and Thomas C. Mettenleiter were involved in conceptualization of the study. Julia Sehl‐Ewert, Theresa Schwaiger, and Julia E. Hölper performed animal experiments. Julia Sehl‐Ewert, Theresa Schwaiger, Alexander Schäfer, and Ulrike Blohm performed data analysis, results interpretation and figures. Julia Sehl‐Ewert, Theresa Schwaiger and Alexander Schäfer wrote the original draft. All authors critically reviewed the manuscript. Thomas C. Mettenleiter, Jens P. Teifke, Barbara G. Klupp, and Ulrike Blohm provided resources. Thomas C. Mettenleiter and Ulrike Blohm supervised the study.

## Supporting information


**FIGURE S1** Gating strategy for the identification of cellular infiltration by flow cytometry. Single cells were identified by consecutive FSC‐A versus FSC‐H and SSC‐A versus SSC‐H gating followed by excluding cellular debris via FSC‐A versus SSC‐A gating. Cells were subdivided into CD45^hi^ and CD45^lo^/CD11b^+^ cells (1). From CD45^hi^ cells, cells were further analyzed based on CD11b and CD3 expression. Granulocytes were identified as CD3^−^CD11b^+^/Ly6G^+^ (2) and monocytes/macrophages were identified as CD3^−^/CD11b^+^/Ly6G^−^ (3). T lymphocytes expressed CD3^+^/CD11b^−^ and were subdivided into CD8^+^ cytotoxic T cells (4) and CD4^+^ T helper cells (5). NK cells were identified as CD3^−^/CD11b^−^/NK1.1^+^ (6) whereas B lymphocytes were distinguished by CD3^−^/CD11b^−^/B220^+^ (7) expression

**FIGURE S2** Viral antigen detection in the temporal lobe of a mouse 49 days pi. The low number of viral antigen positive neurons is indicated (arrow), immunohistochemistry, polyclonal rabbit antibody against PrV glycoprotein B, ABC‐method, magnification 20x

**FIGURE S3** Neuronal necrosis in the temporal lobe. (A) Semiquantitative scoring of neuronal necrosis at different time point post infection. (B and C) Representative temporal lobe section of a mouse 12 days pi showing lymphohistiocytic meningoencephalitis with multifocal necrotic neurons (arrows) as well as mild perivascular edema (arrowhead), hematoxylin and eosin stain, magnification 20x (B) and 40x (C)

**FIGURE S4** CD79^+^ B lymphocytic infiltration of the temporal lobe (TL) 21 days pi, immunohistochemistry, monoclonal mouse anti‐human CD79^+^ antibody, ABC method, magnification 20x

**FIGURE S5** GFAP^+^ astrocyte immunostaining of the temporal lobe (TL). In contrast to a mock‐infected animal, mild astrocytosis is present in infected mice at day 21 pi bordering parenchymal and perivascular lesions, immunohistochemistry, polyclonal rabbit anti‐bovine, ABC‐method, magnification 10x

## Data Availability

The data that support the findings of this study are available on request from the corresponding author.
