# Supplementary figures and images for: Clinical, neuropathological, and immunological short‐ and long‐term feature of a mouse model mimicking human herpes virus encephalitis
Source: Brain Pathol. 2021 Oct 28;32(3):e13031. doi: 10.1111/bpa.13031 (PMC9048517; doi:10.1111/bpa.13031)

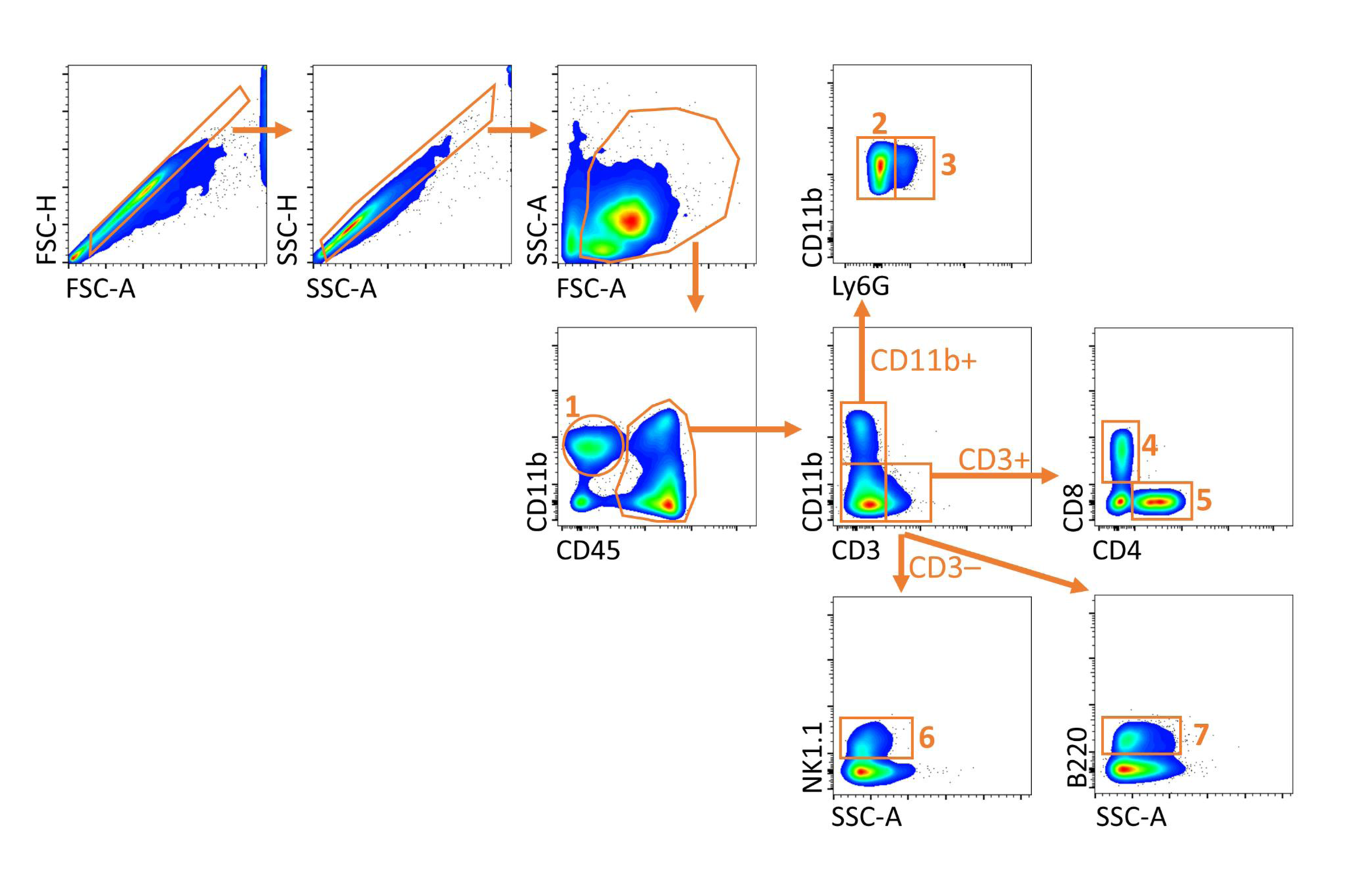

Supplement: Supplementary file 1 — FIGURE S1 Gating strategy for the identification of cellular infiltration by flow cytometry. Single cells were identified by consecutive FSC‐A versus FSC‐H and SSC‐A versus SSC‐H gating followed by excluding cellular debris via FSC‐A versus SSC‐A gating. Cells were subdivided into CD45hi and CD45lo/CD11b+ cells (1). From CD45hi cells, cells were further analyzed based on CD11b and CD3 expression. Granulocytes were identified as CD3−CD11b+/Ly6G+ (2) and monocytes/macrophages were identified as CD3−/CD11b+/Ly6G− (3). T lymphocytes expressed CD3+/CD11b− and were subdivided into CD8+ cytotoxic T cells (4) and CD4+ T helper cells (5). NK cells were identified as CD3−/CD11b−/NK1.1+ (6) whereas B lymphocytes were distinguished by CD3−/CD11b−/B220+ (7) expression [file BPA-32-e13031-s003.tif]

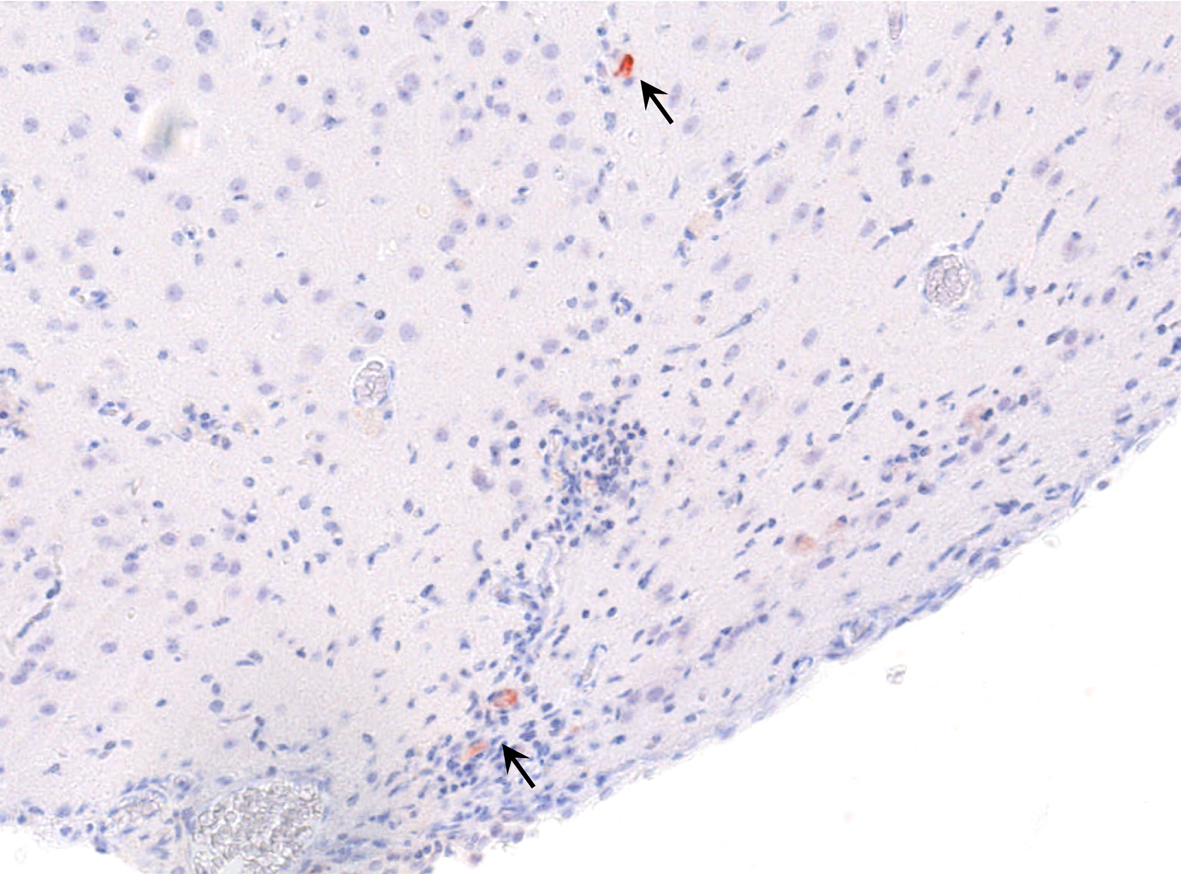

Supplement: Supplementary file 2 — FIGURE S2 Viral antigen detection in the temporal lobe of a mouse 49 days pi. The low number of viral antigen positive neurons is indicated (arrow), immunohistochemistry, polyclonal rabbit antibody against PrV glycoprotein B, ABC‐method, magnification 20x [file BPA-32-e13031-s001.tif]

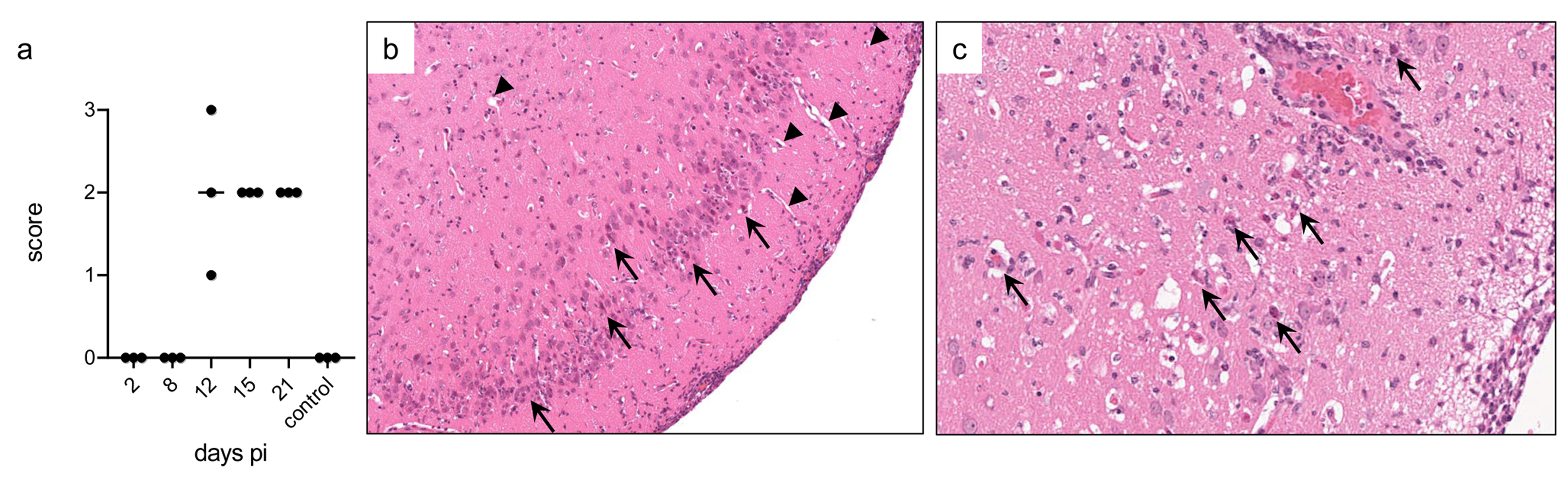

Supplement: Supplementary file 3 — FIGURE S3 Neuronal necrosis in the temporal lobe. (A) Semiquantitative scoring of neuronal necrosis at different time point post infection. (B and C) Representative temporal lobe section of a mouse 12 days pi showing lymphohistiocytic meningoencephalitis with multifocal necrotic neurons (arrows) as well as mild perivascular edema (arrowhead), hematoxylin and eosin stain, magnification 20x (B) and 40x (C) [file BPA-32-e13031-s005.tif]

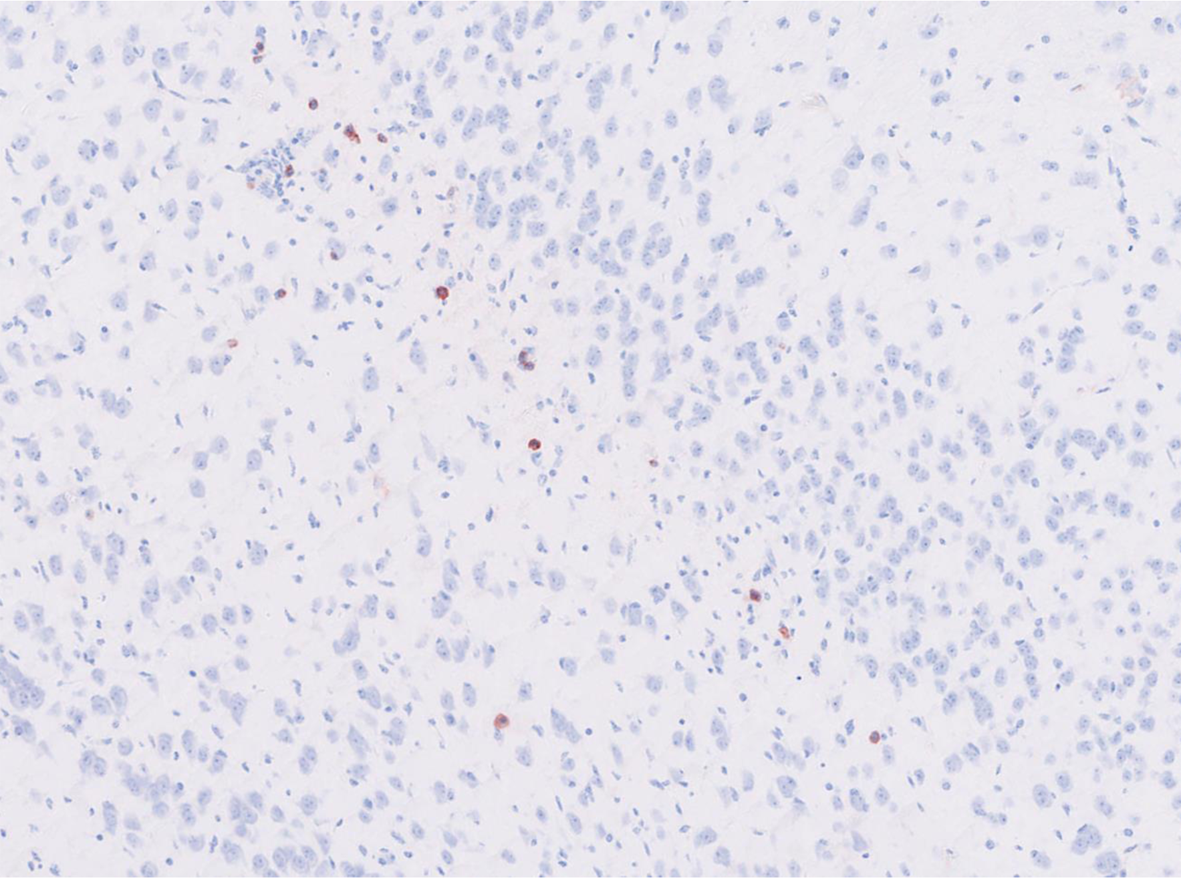

Supplement: Supplementary file 4 — FIGURE S4 CD79+ B lymphocytic infiltration of the temporal lobe (TL) 21 days pi, immunohistochemistry, monoclonal mouse anti‐human CD79+ antibody, ABC method, magnification 20x [file BPA-32-e13031-s004.tif]

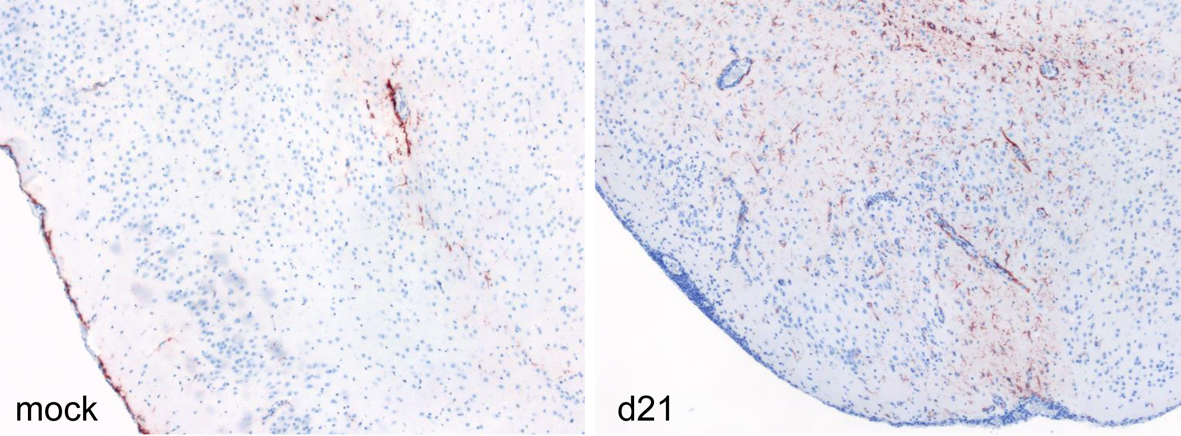

Supplement: Supplementary file 5 — FIGURE S5 GFAP+ astrocyte immunostaining of the temporal lobe (TL). In contrast to a mock‐infected animal, mild astrocytosis is present in infected mice at day 21 pi bordering parenchymal and perivascular lesions, immunohistochemistry, polyclonal rabbit anti‐bovine, ABC‐method, magnification 10x [file BPA-32-e13031-s002.tif]
